# FGF-independent MEK1/2 signalling in the developing foetal testis is essential for male germline differentiation in mice

**DOI:** 10.1186/s12915-023-01777-x

**Published:** 2023-12-05

**Authors:** Rheannon O. Blücher, Rachel S. Lim, Ellen G. Jarred, Matthew E. Ritchie, Patrick S. Western

**Affiliations:** 1 Centre for Reproductive Health, Hudson Institute of Medical Research and Department of Molecular and Translational Science, Monash University, Clayton, VIC 3168 Australia; 2 Epigenetics and Development Division, The Walter and Eliza Hall Institute of Medical Research, Parkville, VIC 3052 Australia

**Keywords:** Germline development, MEK1/2, FGF9, Testis, Foetal gonad, Sex differentiation, Germ cell, Germline mitotic arrest, MAP kinase

## Abstract

**Background:**

Disrupted germline differentiation or compromised testis development can lead to subfertility or infertility and are strongly associated with testis cancer in humans. In mice, SRY and SOX9 induce expression of *Fgf9*, which promotes Sertoli cell differentiation and testis development. FGF9 is also thought to promote male germline differentiation but the mechanism is unknown. FGFs typically signal through mitogen-activated protein kinases (MAPKs) to phosphorylate ERK1/2 (pERK1/2). We explored whether FGF9 regulates male germline development through MAPK by inhibiting either FGF or MEK1/2 signalling in the foetal testis immediately after gonadal sex determination and testis cord formation, but prior to male germline commitment.

**Results:**

pERK1/2 was detected in Sertoli cells and inhibition of MEK1/2 reduced Sertoli cell proliferation and organisation and resulted in some germ cells localised outside of the testis cords. While pERK1/2 was not detected in germ cells, inhibition of MEK1/2 after somatic sex determination profoundly disrupted germ cell mitotic arrest, dysregulated a broad range of male germline development genes and prevented the upregulation of key male germline markers, DPPA4 and DNMT3L. In contrast, while FGF inhibition reduced Sertoli cell proliferation, expression of male germline markers was unaffected and germ cells entered mitotic arrest normally. While male germline differentiation was not disrupted by FGF inhibition, a range of stem cell and cancer-associated genes were commonly altered after 24 h of FGF or MEK1/2 inhibition, including genes involved in the maintenance of germline stem cells, Nodal signalling, proliferation, and germline cancer.

**Conclusions:**

Together, these data demonstrate a novel role for MEK1/2 signalling during testis development that is essential for male germline differentiation, but indicate a more limited role for FGF signalling. Our data indicate that additional ligands are likely to act through MEK1/2 to promote male germline differentiation and highlight a need for further mechanistic understanding of male germline development.

**Supplementary Information:**

The online version contains supplementary material available at 10.1186/s12915-023-01777-x.

## Background

Development of functional sperm and oocytes is essential for fertility and transmission of genetic and epigenetic information to offspring. A critical part of germline development involves the commitment of germ cells to male or female development in response to somatic cell signalling [1]. Germ cells within a developing testis commit to male development from embryonic day (E)12.5 and enter mitotic arrest between E13.5 and E15.5 [1, 2]. They then re-enter the cell cycle and establish spermatogonial stem cells (SSCs) before entering meiosis during spermatogenesis in post-natal life. In contrast, commitment to female germline in the ovary is closely followed by entry into meiosis by E15.5 [1–3].

Male germline differentiation depends on development of an appropriate testicular environment. In XY mice, *Sry* (sex determining region Y) and *Sox9* (SRY box 9) drive pre-supporting cell commitment to Sertoli cell development at E11.5 [4, 5]. Sertoli cells form testis cords that enclose germ cells, a process supported by FGF9 (fibroblast growth factor 9), which further induces *Sox9* expression and drives Sertoli cell proliferation in conjunction with PGD2 (prostaglandin D_2_) [1, 6–8]. *Fgf9* expression peaks at E11.5, before declining to low levels by E13.5 [6, 7, 9–11]. Loss of function mutations in *Fgf9* or its receptor, *Fgfr2*, disrupt testis development leading to ovary or ovotestis formation, reduced germ cell numbers and germline sex reversal [6, 7, 12–14].

Evidence suggests that FGF9 also directly promotes male germline development by acting as a meiosis inhibiting factor via repressing *Stra8* (stimulated by retinoic acid gene 8) and ensuring expression of male germline genes including *Nanos2* (nanos C2HC-type zinc finger 2) and *Dnmt3l* (DNA methyltransferase 3 like) [9, 15]. However, male germ cells develop in *Wnt4/Fgf9* double null mice [16], indicating that *Fgf9* is dispensable for male germline differentiation in this double null background. Furthermore, the mechanism through which FGF9 promotes male germ cell development in wild type mice remains unknown.

Fibroblast growth factor (FGF) ligands, including FGF9, are also required for the establishment of pluripotent embryonic germ cells (EGCs) from primordial germ cells (PGCs) in culture and promote proliferation and an undifferentiated state in SSCs [17–19]. Consistent with this, ectopic FGF9 maintained germ cell expression of the pluripotency genes *Sox2* and *Oct4* in XY gonad cultures [9, 20]. Moreover, isolated E11.5 or E12.5 XY germ cells exposed to high levels of FGF9 maintained proliferation, but low levels of FGF9 promoted male germ cell differentiation [21]. *Fgf9* is downregulated by E13.5 and the potential for germ cells to make EGCs is lost from E12.5, consistent with germ cell entry into mitotic arrest and repression of pluripotency between E13.5 and E15.5 [9, 22, 23].

FGF ligands bind FGF receptors (FGFRs) and rapidly activate intracellular responses via mitogen-activated protein kinase (MAPK) signalling, including through MEK1/2 and phosphorylation of its target ERK1/2 (extracellular signal-regulated kinase 1/2) [24–26]. Low levels of phosphorylated ERK1/2 (pERK1/2) were detected in isolated E11.5 XX and XY germ cells, but gradually increased between E12.5 and E14.5 [27], highlighting a potential role for MEK1/2 signalling during foetal germ cell development. Moreover, inhibiting MEK1/2-ERK1/2 signalling maintains stem cells in ground state pluripotency [28], demonstrating a role for pERK1/2 in priming differentiation of pluripotent cells, an activity facilitated by the E26 transformation-specific (ETS) factors, ETV4 and ETV5 [29, 30].

Germline developmental outcomes in previous studies that have deleted *Fgf9* or *Fgfr2* or manipulated FGF signalling are potentially complicated by somatic sex reversal mediated by loss of FGF signalling, or by disrupted male germline differentiation and survival caused by isolation of germ cells from the somatic environment. In this study, we hypothesised that FGF9 signalling through MEK1/2 promotes male germline development soon after supporting cells commit to Sertoli cell development and testis differentiation. We inhibited FGF receptor or MEK1/2 signalling at E12.5 in intact gonads, after commitment to testis development at E11.5 but before male germline differentiation and entry to mitotic arrest. Our data demonstrate that inhibiting MEK1/2 signalling at E12.5 compromised Sertoli cell proliferation and organisation in the absence of sex reversal, disrupted germ cell mitotic arrest and substantially dysregulated male germline differentiation. Inhibiting FGFR compromised Sertoli cell proliferation in the absence of sex reversal, but male germ cell differentiation proceeded normally. Our data indicate that XY germ cells require FGF-independent MEK1/2 signalling to successfully mediate male germline differentiation.

## Results

### Inhibition of FGF or MEK1/2 signalling disrupts testis development

Loss of *Fgf9* in the developing testis leads to somatic sex reversal and consequent germline sex reversal. To avoid this significant confounding factor, we allowed sex determination to occur before inhibiting FGFR or MEK1/2 and assessed how this affected male germline differentiation in an intact gonad. This allowed us to temporally separate somatic sex determination from male-specific germline differentiation. To achieve this, E12.5 XY gonad-mesonephros samples were cultured with vehicle control (DMSO) or a range of small molecule inhibitors of FGFR1-3, MEK1/2, p38MAPK, and PI3K to screen for germline developmental effects mediated by these signalling pathways. Inhibitors initially used included pan-FGFR (FGFR1-3) inhibitor BGJ398/Infigratinib (FGFRi; IC50 ~ 1.0 nM), MEK1/2 inhibitor PD0325901/Mirdametinib (MEKi; IC50 0.33 nM), p38MAPK inhibitor PH-797804 (p38i; IC50 26 nM) or PI3K inhibitor GSK1059615 (PI3Ki, IC50 5 nM). Importantly, all of these inhibitors have IC50 values in the low nM range and have been extensively validated in clinical trials demonstrating their high specificity, potency and cell tolerance (Table 1).

**Table 1:** Summary of treatments and doses used in gonad cultures

Treatment	Dose	IC50 and target	Clinical trial phase, ClinicalTrials.gov identifier	Supplier, catalogue number	References
DMSO (vehicle control)	Equal to DMSO in drug treatments (≥ 1/5000)	NA	NA	Thermo-Fisher, D12345	
BGJ398/ Infigratinib (FGFRi)	125, 250, 500, 1000 and 2500 nM	IC50 – FGFR1–3: 0.9–1.4 nM, FGFR4: 60 nM	Phase II, NCT02150967	SelleckChem, S2183	[31]
PD0325901/ Mirdametinib (MEKi)	125, 250, 500 and 1000 nM	IC50 – MEK: 0.33 nM	Phase II, NCT03962543	SelleckChem, S1036 or MedChem Express, HY-10254	[32]
PH-797804 (p38i)	500 nM	IC50 – p38α: 26 nM; p38β 102 nM	Phase II, NCT00559910	MedChem Express, HY-10403	[33]
GSK1059615 (PI3Ki)	500 nM	IC50 – PI3Kα/ β/δ/γ: 0.4–5 nM, mTOR: 12 nM	Phase I, NCT00695448	MedChem Express, HY-12036	
Recombinant mouse FGF9	50 ng/mL	FGFR1-3	NA	R&D systems, 7399-F9-025	[20]
AZD4547 (second FGFR inhibitor)	125, 250 and 500 nM	IC50 – FGFR1-3: 0.2–2.5 nM; FGFR4: 165 nM; VEGFR2: 24 nM	Phase II, NCT02465060	SelleckChem, S2801	[34]
SU5402 (third FGFR inhibitor)	5000 nM	IC50 VEGFR: 20 nM; FGFR1: 30 nM	N/A	MedChem Express, HY-10407	[35]
Ralimetinib dimesylate/ LY2228820 (second p38 inhibitor)	500 nM	IC50 – p38α: 5.3 nM; p38β: 5.3 nM	Phase I, NCT01663857	MedChem Express, HY-13241	[36]
PF-04691502 (second PI3K inhibitor)	500 nM	IC50 – PI3Kα/ β/δ/γ: 1.6–2.1 nM; mTOR: 16 nM	Phase II, NCT01420081	MedChem Express, HY-15177	[37]

**Fig. 1:**
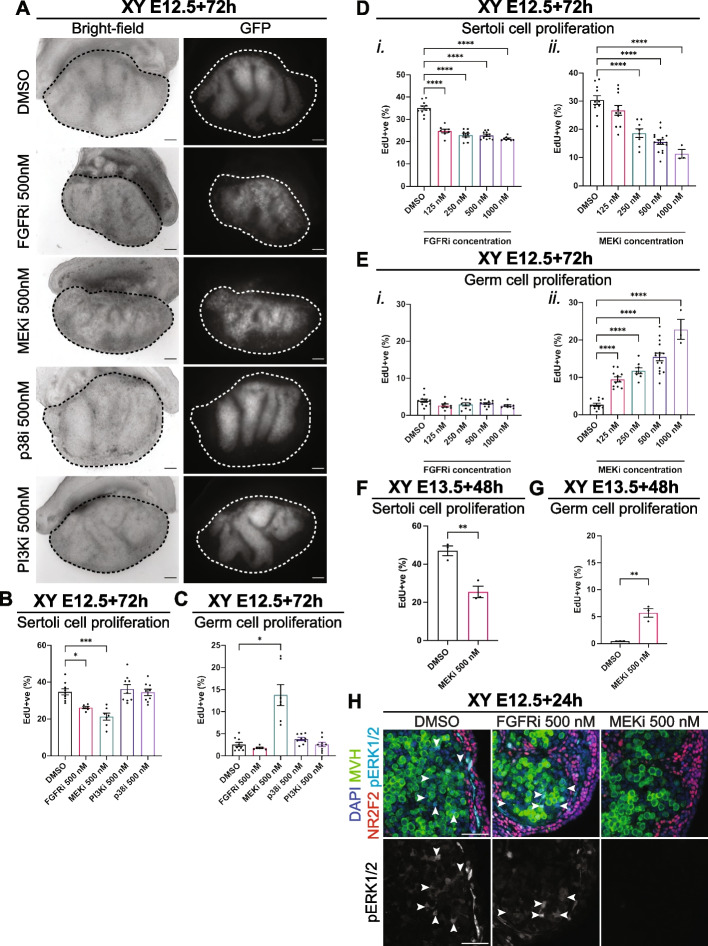
FGF and MEK1/2 inhibition disrupts foetal testis development but only MEK1/2 inhibition disrupts germ cell mitotic arrest. **A** Bright-field and GFP images of E12.5 XY gonad-mesonephros tissue cultured for 72 h with DMSO, or 500 nM of FGFRi, MEKi, p38i or PI3Ki. Scale bar: 100 μm. Dotted lines highlight the gonad. **B–E** Flow cytometric analysis of Sertoli (**B**,**D**) or germ (**C**,**E**) cell proliferation based on EdU incorporation in XY E12.5 gonad-mesonephros tissue cultured for 72 h with DMSO, 500 nM of FGFRi, MEKi, PI3Ki or p38i (**B**,**C**) or 125, 250, 500 or 1000 nM of FGFRi or MEKi (**D**,**E**). **F**,**G** Flow cytometric analysis of Sertoli (**F**) or germ (**G**) cell proliferation based on EdU incorporation in XY E13.5 gonad-mesonephros tissue cultured for 48 h with DMSO or 500 nM of FGFRi or MEKi. **H** Immunofluorescent images of E12.5 gonad-mesonephros tissue cultured for 24 h with DMSO, 500 nM of FGFRi or MEKi demonstrating MEK1/2 signalling activity. Top panel: DAPI (blue), MVH (green), NR2F2 (red), pERK1/2 (cyan). Bottom panel: pERK1/2 (grey). Scalebar represents 50 μm. Replicates: **A–C** *n* = 6–9, **D**,**E** *n* = 3–16, **F**,**G** *n* = 3, **H** *n* = 3–4. Statistics: **B**, **D**, **E** Ordinary one-way ANOVA with Tukey’s multiple comparison, **C** Brown-Forsythe and Welch ANOVA with Dunnett’s T3 multiple comparisons, **F**,**G** Unpaired two-tailed *t*-test. Error bars: Mean ± SEM. Significance between control and treatment: *P < 0.05, **P < 0.01, ***P < 0.001, ****P < 0.0001

To account for the difference between cell free IC50 values and drug bioavailability in gonad culture, we initially used a starting concentration of 500 nM for each drug. This is consistent with our observation that drugs with similar IC50s maximally inhibit their targets in gonad cultures in the 100–1000 nM range. The vehicle control, DMSO, was used at a dilution of ≥ 1/5000 in all experiments, a concentration that does not affect gonad or germline development [20, 38]. Bright-field and fluorescence examination of E12.5 testis-mesonephros samples cultured with control (DMSO) or drug for 72 h provided an initial readout of the impact of each drug based on germ cell organisation within testis cords, marked by germ cell-specific expression of *Oct4-eGFP* (Fig. 1A). DMSO controls developed well-defined cords containing germ cells, but FGFRi and MEKi resulted in poor testis cord structure and some GFP-positive germ cells were located outside the testis cords. p38i- and PI3Ki-treated gonads were morphologically similar to DMSO controls, with GFP-positive germ cells contained within well-defined testis cords (Fig. 1A).

To further determine the effects of each treatment on testis development, we added 5-ethynyl-2’-deoxyuridine (EdU) during the final 2 h of culture and used flow cytometry to assess proliferation based on EdU incorporation during S-phase and DNA content using propidium iodide (PI) staining in E12.5 gonad-mesonephric samples cultured for 72 h with each inhibitor [20, 38, 39] (Fig. 1B,C). As expected, Sertoli cells were highly proliferative in DMSO controls (Fig. 1B). However, FGFRi or MEKi reduced Sertoli cell proliferation compared to XY DMSO controls (*P* < 0.05 or *P* < 0.001, respectively, Fig. 1B). As expected, germ cell proliferation was very low in DMSO controls (Fig. 1C), confirming that the germ cells had entered mitotic arrest, a key milestone in male germline differentiation [2]. In contrast, germ cell proliferation was substantially higher in MEKi-treated gonads (*P* < 0.05), demonstrating that germ cell mitotic arrest was disrupted (Fig. 1C). However, the percentage of germ cells incorporating EdU in FGFRi-treated testes was similar to DMSO controls, demonstrating that the germ cells had entered mitotic arrest (Fig. 1C). As FGFRi was expected to disrupt germ cell mitotic arrest, this outcome was confirmed using another potent FGFR inhibitor (AZD4547; Table 1), which also resulted in reduced Sertoli cell proliferation but did not disrupt germ cell mitotic arrest (Additional file 1: Fig. S1A,B). Neither p38i (PH-797804) nor PI3Ki (GSK1059615) affected Sertoli cell proliferation or germ cell mitotic arrest (Fig. 1B,C), an outcome confirmed using independent, p38 and PI3K inhibitors, ralimetanib dimesylate (LY2228820) and PF-04691502 (Table 1; Additional file 1: Fig. S1C,D).

Given both FGFRi and MEKi reduced Sertoli cell proliferation and disrupted testis cord development, we analysed AMH, SOX9 and FOXL2 using immunofluorescence (IF) to ensure that FGFR or MEK inhibition did not result in somatic sex reversal. Notably, robust SOX9 and AMH staining was detected by IF in male DMSO, FGFRi and MEKi samples but not in female gonads (Additional file 2: Fig. S2A), indicating that treated XY gonads maintained a male phenotype. Consistent with this, assessment of SOX9 intensity using flow cytometry demonstrated that SOX9 expression was not reduced in FGFRi- or MEKi-treated samples compared to DMSO controls (Additional file 2: Fig. S2B). Furthermore, IF staining for the female marker, FOXL2, revealed strong expression in female gonads, but minimal staining in male DMSO, FGFRi or MEKi samples, although occasional FOXL2-positive cells were detected in MEKi-treated samples (Additional file 2: Fig. S2C). Importantly, these data demonstrate that inhibition of FGFR or MEK1/2 at E12.5 did not result in somatic sex reversal. This allowed us to further assess the effects of FGF or MEK1/2 inhibition in the environment of a developing testis on male germline differentiation and in the absence of somatic sex reversal.

### FGF and MEK1/2 signalling are both required for Sertoli cell proliferation, but only MEK1/2 is required for germ cell mitotic arrest

To titrate the dose response to MEK1/2 and FGFR inhibition, E12.5 XY gonad-mesonephros samples were cultured with MEKi or FGFRi at 0 (DMSO diluted ≥ 1/5000), 125, 250, 500 and 1000 nM and assessed using flow cytometry (Fig. 1D,E and Additional file 3: Fig. S3). Compared to DMSO control, proliferation of SOX9 expressing Sertoli cells was reduced by all doses of FGFRi ≥ 125 nM (*P* < 0.0001; Fig. 1Di) and ≥ 250 nM MEKi (*P* < 0.0001, Fig. 1Dii). Of interest, although the maximal impact of FGFRi on Sertoli cell proliferation occurred at 125 nM, it did not further reduce Sertoli cell proliferation even at 1000 nM and this effect was noticeably less than that of MEKi at doses of 500 and 1000 nM (Fig. 1Di vs Dii). Based on these data, doses of 500 nM of FGFRi and 500 nM of MEKi were used for further experiments.

Consistent with our initial observations (Fig. 1C), although Sertoli cell proliferation was reduced, germ cell mitotic arrest remained unaffected by FGFRi even with a dose of 1000 nM (Fig. 1Ei). To confirm this outcome, we tested whether a very high dose of 2500 nM FGFRi, which is ~ 2500 × the IC50 value (Table 1) and 20 × the 125 nM minimal dose affecting Sertoli cell proliferation (Fig. 1Di), might affect germ cell mitotic arrest. However, this again resulted in a similar reduction in Sertoli cell proliferation, but no effect on germ cell mitotic arrest (Additional file 1: Fig. S1E-F). We next compared outcomes for FGFRi and AZD4547 with and third inhibitor, SU5402, which was previously used at 5000 nM to inhibit FGFR [9]. Confirming the outcomes obtained using FGFRi and AZD4547, 5000 nM SU5402 reduced Sertoli cell proliferation to a similar extent as FGFRi and AZD4547, but it did not affect germ cell mitotic arrest (Table 1; Additional file 1: Fig. S1E-F). In contrast, MEKi potently disrupted germ cell mitotic arrest even when used at 125 nM, with increasingly high proportions of EdU-positive proliferative germ cells as MEKi concentration increased (*P* < 0.0001, Fig. 1Eii).

In repeated experiments, MEK1/2 inhibition profoundly disrupted germ cell mitotic arrest in E12.5 XY gonads but three different FGFR inhibitors did not, even though FGFs typically elicit a response through MEK1/2-pERK1/2 within 10–15 min [25, 26]. As FGF9 expression peaks at E11.5 [11], a potential explanation for the inability of FGFRi to disrupt germ cell mitotic arrest could be that FGF9 was inhibited too late in E12.5 cultures. However, as *Fgf9* or *Fgfr2* genetic deletions cause somatic sex reversal [6, 7, 12–14], inhibition of FGFR at E11.5 is expected to cause somatic sex reversal and consequent germ cell sex reversal that would substantially confound data interpretation. To avoid the confounding effect of male to female sex reversal and provide a clearer outcome for the study, we did not include studies of FGFR inhibition at E11.5.

To determine if MEK1/2 inhibition affected Sertoli cell proliferation and germ cell mitotic arrest after E13.5, E13.5 XY gonad-mesonephros samples were cultured for 48 h with DMSO or 500 nM MEKi. MEKi treatment from E13.5 significantly reduced Sertoli (*P* < 0.01, Fig. 1F) and increased germ cell proliferation (*P* < 0.01, Fig. 1G). However, the effect of MEKi on germ cell mitotic arrest was diminished compared to E12.5 (E13.5: EdU 6% vs E12.5: 15%, *P* < 0.0001 E12.5 MEKi vs E13.5 MEKi; Fig. 1Eii,G), indicating that the ability of MEKi to disrupt mitotic arrest decreased between E12.5 and E13.5.

We previously demonstrated that FGF9 induces proliferation of XX somatic cells at rates similar to XY gonads [20]. To confirm that FGFRi and MEKi effectively blocked FGF9 activity, XX E12.5 gonads were cultured for 48 h in media containing DMSO, 50 ng/mL FGF9, 500 nM FGFRi, 500 nM MEKi, FGF9 + FGFRi or FGF9 + MEKi and assessed using flow cytometry. As expected, FGF9 substantially increased the proliferation of XX gonadal somatic cells compared to XX controls (*P* < 0.05,* P* < 0.0001 ; Additional file 1: Fig. S1G). Critically, both FGFRi and MEKi completely neutralised FGF9, with somatic cell proliferation decreased to XX control levels in FGF9 + FGFRi or FGF9 + MEKi-treated XX gonads (Additional file 1: Fig. S1Gi,Gii). In contrast, neither p38i nor PI3Ki counteracted FGF9-induced somatic cell proliferation in XX gonads, indicating that neither p38MAPK nor PI3K regulate primary pathways through which FGF drives somatic cell proliferation in developing gonads (Additional file 1: Fig. S1Giii).

### Inhibition of MEK1/2 completely abolished ERK1/2 phosphorylation in the developing testis, but FGFR inhibition did not

FGF activation of MEK1/2 rapidly results in phosphorylation of ERK1/2, and MEK1/2 inhibition blocks this activity [25, 26]. To determine if inhibition of FGF or MEK1/2 signalling prevented phosphorylation of ERK1/2, E12.5 XY gonad-mesonephros samples were cultured with 500 nM FGFRi or MEKi for 24 h and pERK1/2 was assessed using IF (Fig. 1H and Additional file 1: Fig. S1H). Surprisingly, pERK1/2 was not detected in MVH (mouse vasa homolog) expressing germ cells in the developing testis. However, consistent with MEK1/2 activity in Sertoli cells, pERK1/2 was detected at low levels in MVH negative somatic cells within testis cords in DMSO controls. While there are no other somatic cells within testis cords, Sertoli cell localisation of pERK1/2 could not be definitively determined using SOX9 IF as the SOX9 and pERK1/2 antibodies were both raised in rabbit. Robust pERK1/2 was also detected in somatic cells outside of the testis cords that appeared to be endothelial cells; however, this was not confirmed. pERK1/2 was not detected in MEKi-treated samples, although it was detected in Sertoli and somatic cells outside of the testis cords in FGFRi-treated gonads (Fig. 1H and Additional file 1: Fig. S1H) demonstrating that MEKi abolished ERK1/2 phosphorylation, but FGFRi did not. Consistent with this, MEKi reduced Sertoli cell proliferation to a greater extent than FGFRi (*P* < 0.0001 MEKi vs FGFRi, Additional file 1: Fig. S1I). However, as 500 nM of FGFRi completely blocked FGF9-induced proliferation in XX somatic cells (Additional file 1: Fig. S1Gi), the most likely explanation for the inability of FGFRi to completely eliminate pERK1/2 in Sertoli cells is that MEK1/2 may be activated independently of FGFR, perhaps by PGD2 [1, 8, 40] or other ligands.

### FGF and MEK1/2 signalling is required for normal testis cord formation

**Fig. 2:**
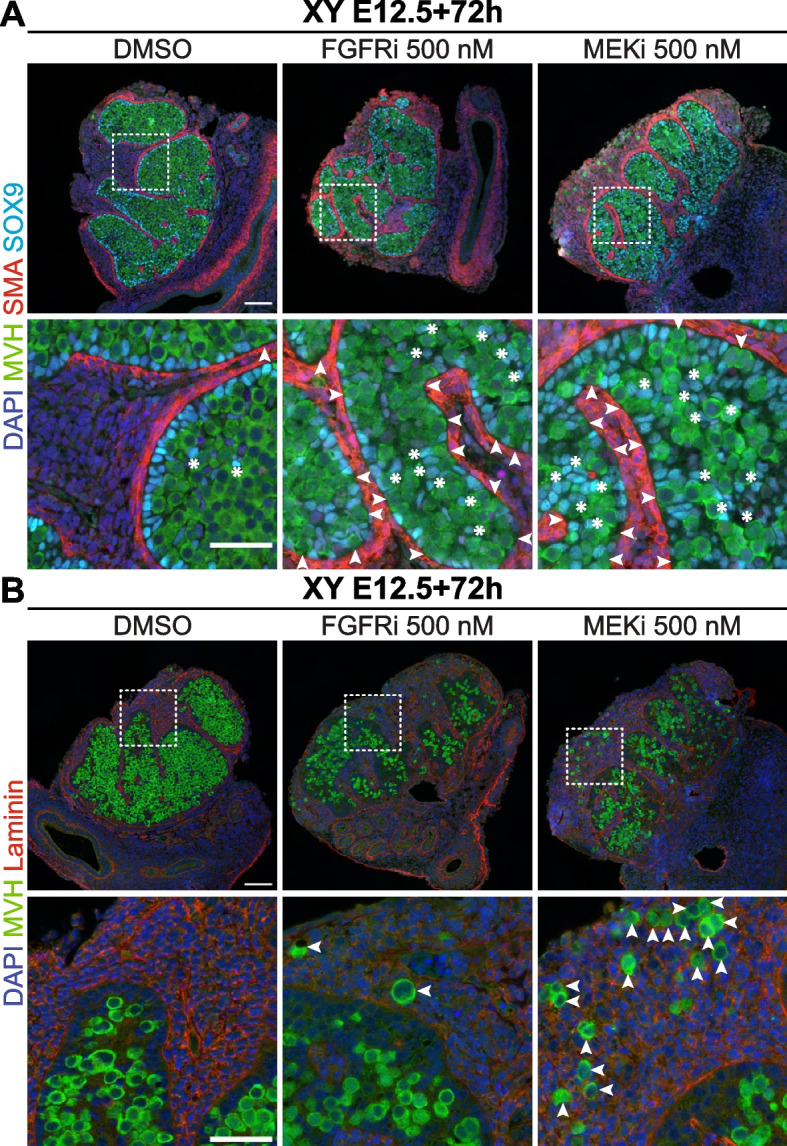
FGF and MEK1/2 signalling is required for normal testis cord formation. Immunofluorescent images of XY E12.5 gonad-mesonephros tissue cultured with DMSO, 500 nM FGFRi or 500 nM MEKi for 72 h showing Sertoli (**A**) and germ (**A**,**B**) cell localisation. DAPI (blue), MVH (green), SMA (red: **A**) or Laminin (red: **B**) and SOX9 (cyan: **A**). Scale bars: top panel 100 μm, bottom panel 50 μm. Replicates: *n* = 3–4. **A** White arrows identify gaps in the Sertoli cell layer at the testis cord basement membrane; white asterisks identify Sertoli cells dispersed within the inner area of the testis cords. **B** White arrows identify germ cells localised outside testis cords

As gonad whole-mount images indicated testis cords were disrupted by FGFRi or MEKi (Fig. 1A), we used IF to investigate MVH-positive germ cells relative to SOX9 expressing Sertoli cells, SMA (smooth muscle actin) expressing peritubular myoid cells or laminin, which delineate testis cords. In DMSO controls, the majority of Sertoli cells were organised in a single layer at the testis cord basement membrane, with germ cells very rarely found outside the cords (Fig. 2). In FGFRi- and MEKi-treated samples, some Sertoli cells localised to the testis cord basement membrane, but gaps were evident between the Sertoli cells, and many Sertoli cells remained dispersed throughout the interior of the testis cords (Fig. 2A). Furthermore, germ cells were occasionally present in the gaps between Sertoli cells at the testis cord basement membrane (Fig. 2A) and were mis-localised outside testis cords in FGFRi-treated cultures, although this was more common in MEKi-treated gonads (Fig. 2B).

### MEK1/2 signalling is required for male germline differentiation

**Fig. 3:**
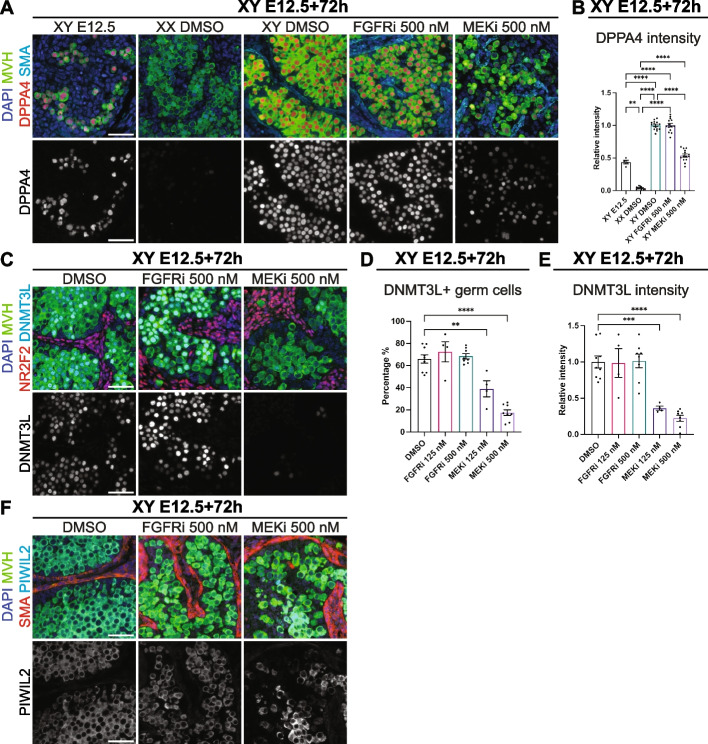
MEK1/2 signalling is required for male germline differentiation. Analysis of E12.5 XY gonad-mesonephros or E12.5 XY or XX gonad-mesonephros cultured for 72 h with DMSO, 125 or 500 nM FGFRi or MEKi. **A** Immunofluorescent images demonstrating DPPA4 localisation. Top panel: DAPI (blue), MVH (green), DPPA4 (red), SMA (cyan). Bottom panel: DPPA4 (grey). **B** DPPA4 staining intensity in germ cells determined by flow cytometry. **C** Immunofluorescent images demonstrating DNMT3L localisation. Top panel: DAPI (blue), MVH (green), NR2F2 (red), DNMT3L (cyan). Bottom panel: DNMT3L (grey). **D**,**E** Percentage DNMT3L-positive germ cells (**D**) and DNMT3L staining intensity (**E**) determined by flow cytometry. **F** Immunofluorescent images demonstrating PIWIL2 localisation. Top panel: DAPI (blue), MVH (green), SMA (red), PIWIL2 (cyan). Bottom panel: PIWIL2 (grey). Scale bars: 50 μm. Replicates: **A**, **C**, **F** *n* = 3–4, **B** *n* = 4–14, **D**,**E** *n* = 4–9. Statistics: **B**,**E** Brown-Forsythe and Welch ANOVA with Dunnett’s T3 multiple comparisons, **D** Ordinary one-way ANOVA with Tukey’s multiple comparison. In **B**,**E**, Intensity is relative to E12.5 + 72 h XY DMSO control sample set at 1.0. Error bars: mean ± SEM. Significance between control and treatment: **P < 0.01, ***P < 0.001, ****P < 0.0001

As MEKi prevented germ cell mitotic arrest, the expression of male germline differentiation markers was assessed using IF and flow cytometry in E12.5 XY gonad-mesonephros samples cultured for 72 h with DMSO, 125 or 500 nM FGFRi or MEKi. DPPA4 is expressed in XX and XY germ cells at E12.5, but is upregulated in XY germ cells and repressed in XX germ cells as they differentiate [20]. As expected, DPPA4 was not detected in germ cells of XX gonads but was detected in germ cells of XY E12.5 gonads cultured for 72 h with DMSO, and fluorescence appeared more intense in XY E12.5 + 72 h than in E12.5 XY germ cells (Fig. 3A and Additional file 4: Fig. S4A). While DPPA4 germ cell levels were similar in XY E12.5 + 72 h DMSO and FGFRi cultures, DPPA4 intensity appeared lower in MEKi-treated samples and comparable to E12.5 XY germ cells (Fig. 3A and Additional file 4: Fig. S4A). Confirming this, flow cytometry revealed that the relative DPPA4 germ cell intensity was 2 × higher in XY E12.5 + 72 h DMSO and FGFRi cultures than in E12.5 XY germ cells (*P* < 0.0001), but DPPA4 was expressed at comparable levels in MEKi-treated samples compared to E12.5 XY germ cells (Fig. 3B).

As germ cell proliferation and DPPA4 levels indicated that male germ cells failed to properly differentiate in MEKi-treated XY gonads, we examined two additional male germline markers, DNMT3L and PIWIL2. IF and flow cytometry revealed that the majority of germ cells were DNMT3L positive in DMSO controls and FGFRi-treated gonads (Fig. 3C–E and Additional file 4: Fig. S4B). In contrast, very few germ cells were DNMT3L positive in MEKi-treated gonads (*P* < 0.01, *P *> 0.0001), and the DNMT3L staining intensity was significantly lower than in the DMSO- or FGFRi-treated samples (*P* < 0.001, *P* < 0.0001; Fig. 3C–E and Additional file 4: Fig. S4B). Similarly, PIWIL2 was expressed at similar levels in germ cells of DMSO- and FGFRi-treated samples but was variable in MEKi-treated samples, with some germ cells staining strongly for PIWIL2 and others negative (Fig. 3F and Additional file 4: Fig. S4C). This was not possible to confirm using flow cytometry because a reliable PIWIL2 flow assay could not be developed.

### MEK1/2 inhibition increased STRA8, but failed to properly induce meiosis in XY germ cells

**Fig. 4:**
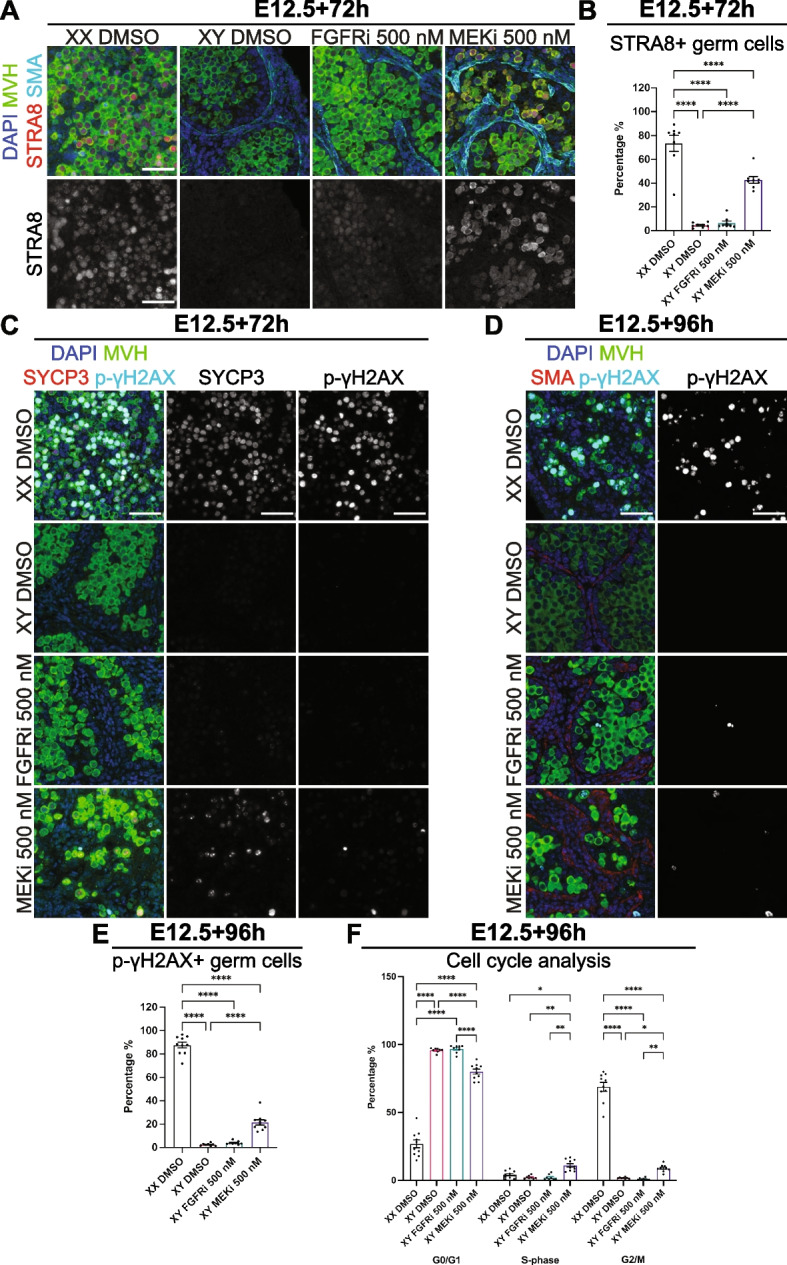
MEK1/2 signalling inhibition permitted *STRA8* expression but failed to effectively induce meiosis in XY germ cells. Analysis of XY or XX E12.5 gonad-mesonephros tissue cultured with DMSO or 500 nM FGFRi or MEKi for 72 h (**A–C**) or 96 h (**D**–**F**). **A** Immunofluorescent images demonstrating STRA8 localisation. Top panel: DAPI (blue), MVH (green), STRA8 (red), SMA (cyan). Bottom panel: STRA8 (grey). **B** Percentage STRA8 positive germ cells determined by flow cytometry. **C**,**D** Immunofluorescent images demonstrating SYCP3 (**C**) and phospho-γH2AX (p-γH2AX) localisation. Left panel: DAPI (blue), MVH (green), SYCP3 (red; **C**) or SMA (red; **D**) and p-γH2AX (cyan). Middle panel: SCP3 (grey; **C**). Right panel: p-γH2AX (grey). **E** Percentage p-γH2AX-positive germ cells determined by flow cytometry. **F** Flow cytometric cell cycle analysis of G0/G1, S-phase and G2/M based on the incorporation of EdU (S-phase) and propidium iodide (DNA content). **A**, **C**, **D** scale bar: 50 μm. Replicates: **A**, **C**, **D** *n* = 3–4, **B** *n* = 8, **E**,**F** *n* = 8–10. Statistics: **B** Ordinary one-way ANOVA with Tukey’s multiple comparison, **E** Brown-Forsythe and Welch ANOVA with Dunnett’s T3 multiple comparisons, **F** Repeated measures two-way ANOVA with Tukey’s multiple comparisons. Error bars: mean ± SEM. Significance between controls and treatment: *P < 0.05, **P < 0.01, ****P < 0.0001

Since MEKi inhibited mitotic arrest and male germline differentiation, the expression of female germline markers was investigated to determine if FGFRi or MEKi induced female development in XY germ cells. As expected, the pre-meiotic marker STRA8 was detected in the germ cells of XX E12.5 + 72 h DMSO-treated gonads but was not detected in XY DMSO controls (Fig. 4A and Additional file 5: Fig. S5A). While some germ cells appeared very weakly positive for STRA8 in FGFRi-treated samples, STRA8-positive germ cells were commonly found in MEKi-treated gonads, particularly in germ cells close to the mesonephric-gonadal boundary (Fig. 4A and Additional file 5: Fig. S5A). However, while STRA8 staining was localised in the germ cell nucleus in XX controls, it was detected in the germ cell cytoplasm and nucleus in MEKi treatments, indicating that nuclear import–export also regulates STRA8 activity (Fig. 4A and Additional file 5: Fig. S5A). Flow cytometry demonstrated that 74% of germ cells were STRA8 positive in XX DMSO controls, but only 5% and 7% were STRA8 positive in XY DMSO control and FGFRi-treated samples (Fig. 4B). The proportion of STRA8-positive germ cells in MEKi-treated gonads was 43%, significantly higher than XY controls (*P* < 0.0001), but lower than XX controls (*P* < 0.0001, Fig. 4B).

To determine whether germ cells in FGFRi- or MEKi-treated XY gonads had entered meiosis, gonad sections were triple stained using antibodies specific for SYCP3 (synaptonemal complex protein 3), phosphorylated γH2AX (p-γH2AX) and MVH (Fig. 4C and Additional file 5: Fig. S5B). SYCP3 and p-γH2AX were detected in most germ cells in 72 h XX DMSO controls but not in XY DMSO- or FGFRi-treated gonads (Fig. 4C and Additional file 5: Fig. S5B). A small number of germ cells were positive for SYCP3 in MEKi-treated gonads and a subset also stained for p-γH2AX (Fig. 4C and Additional file 5: Fig. S5B). In addition, rare cells positive for p-γH2AX were detected in XY control, FGFRi- and MEKi-treated gonads (Additional file 5: Fig. S5B), however, most did not express MVH and were likely to be apoptotic somatic cells in which p-γH2AX also marks double strand DNA breaks.

To test the possibility that meiotic entry of germ cells in FGFRi- or MEKi-treated gonads was delayed, we cultured E12.5 XX and XY gonad-mesonephros samples for 96 h with DMSO or 500 nM FGFRi or MEKi. IF staining revealed that most XX DMSO germ cells were p-γH2AX positive, indicating they had entered meiosis (Fig. 4D and Additional file 5: Fig. S5C). p-γH2AX-positive germ cells were rarely detected in XY DMSO- or FGFRi-treated gonads but were more common in MEKi-treated samples (Fig. 4D and Additional file 5: Fig. S5C). Quantification using flow cytometry revealed that 88% of germ cells were p-γH2AX positive in XX DMSO samples while only 2%, 4% and 21% were p-γH2AX positive in XY control, FGFRi- and MEKi-treated samples, respectively (Fig. 4E).

Cell cycle analysis of germ cells from the same gonads using EdU (S-phase) to quantify DNA synthesis and PI to measure DNA content demonstrated that the majority of germ cells in E12.5 + 96 h cultures were in G2/M in XX DMSO controls, but were in G0/G1 in XY DMSO- and FGFRi-treated gonads (Fig. 4F). Significantly more germ cells were in G2/M in MEKi than in XY DMSO control (*P* < 0.05) or FGFRi cultures (*P* < 0.01), but remained less than in XX controls (*P* < 0.0001, Fig. 4F). Therefore, while MEKi treatment resulted in a significantly greater percentage of p-γH2AX expressing germ cells that were in G2/M, this proportion was substantially lower than in XX controls indicating that meiosis was not properly induced within the normal temporal window following MEK1/2 inhibition.

### The majority of transcriptional divergence occurs after E12.5 in XY and XX germ cells

**Fig. 5:**
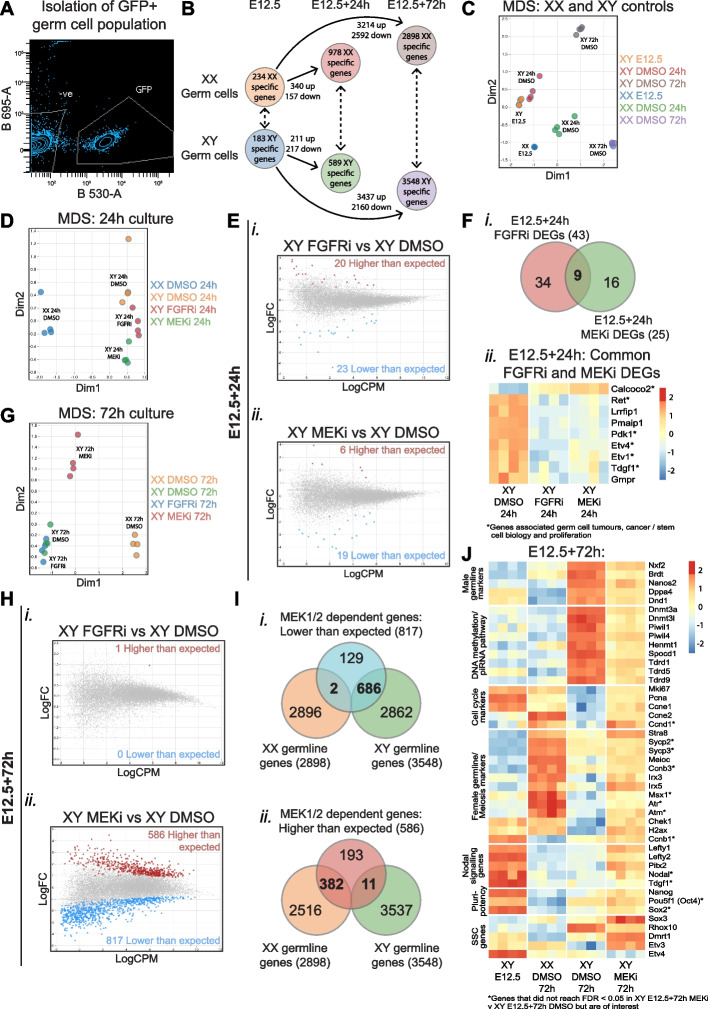
FGF-MEK1/2 signalling supports expression of stem cell-associated genes in early germ cells, but only MEK1/2 signalling is required for male germline differentiation. RNA sequencing analysis of germ cells from XX or XY E12.5 gonads, or XX or XY E12.5 gonads cultured for 24 or 72 h with DMSO or 500 nM FGFRi or MEKi. **A** Example of FACS scatterplot depicting GFP-positive germ cell isolation. **B** Number of differentially expressed genes (DEGs) between XX or XY E12.5 (time 0) and XX or XY DMSO controls from 24 and 72 h cultures. **C** Multidimensional scaling (MDS) of all control conditions. **D** MDS of XX and XY gonads cultured for 24 h. **E** Differential gene expression analysis of XY E12.5 + 24 h FGFRi XY (*i*) or XY E12.5 + 24 h MEKi (*ii*) vs XY E12.5 + 24 h DMSO. **F** Venn diagram of 24 h FGFRi and MEKi DEGs (*i*) and heatmap of common DEGs (*ii*). Asterisks represent genes associated with germ cell tumours, cancer/stem cell biology and/or proliferation. **G** MDS of XX and XY gonads cultured for 72 h. **H** Differential gene expression analysis of XY E12.5 + 72 h FGFRi (*i*) or XY E12.5 + 72 h MEKi (*ii*) vs XY E12.5 + 72 h DMSO. **I** Venn diagram comparing MEKi 72 h culture DEGs expressed lower (*i*) or higher (*ii*) than expected with XX or XY specific genes identified in B. **J** Heatmap of DEGs identified in XY E12.5 + 72 h MEKi vs XY E12.5 + 72 h DMSO associated with male germline differentiation, DNA methylation/piRNA pathway, cell cycle, female germline differentiation/meiosis, Nodal signalling, pluripotency and spermatogonial stem cells (SSCs). Asterisks highlight genes associated with cell cycle, meiosis and pluripotency but are not differentially expressed. For all comparisons, genes with FDR < 0.05 and |logFC|> 0.585 (equivalent to |FC|> 1.5) were considered differentially expressed

We next used RNA sequencing to gain greater insight into genome-wide transcriptional changes in fluorescent activated cell sorting (FACS) isolated *Oct4*-eGFP-positive germ cells of E12.5 XX and XY gonads (Time 0 controls) and gonads cultured for 24 and 72 h with DMSO, FGFRi and MEKi (Fig. 5A). Differential expression analysis identified 183 and 234 genes that were expressed higher in XY and XX germ cells at E12.5, respectively (time 0; false discovery rate (FDR) < 0.05; absolute fold-change (FC) ≥ 1.5, absolute logFC ≥ 0.585; Fig. 5B, Additional file 6: Table S1.1–1.2). Included in the differentially expressed genes (DEGs) that were higher in E12.5 XY germ cells were a range of Nodal signalling associated genes, including *Nodal, Tdgf1 (Cripto), Lefty1*, *Lefty2, Pitx2* and *Otx2*, which are known to be high in XY germ cells at this time point [38, 41–43] (Additional file 6: Table S1.1). E12.5 XX germ cells expressed higher levels of BMP target genes, including *Msx1, Msx2, Id1, Id2, Id3, Stra8* and *Gata2*, consistent with observations that BMP2 promotes female germline development [43, 44] (Additional file 6: Table S1.2). However, despite these sex-specific transcriptional differences in E12.5 germ cells, our functional data strongly indicated that these differences were insufficient to ensure male germline commitment as MEK1/2 inhibition at E12.5 substantially disrupted male germline differentiation (Figs. 1 and 3).

To identify male and female transcriptional changes that occurred as a normal part of sex-specific germline differentiation, XX and XY germ cells of DMSO control samples were compared after 24 and 72 h of culture. Multidimensional scaling (MDS) revealed that although time 0 XY and XX E12.5 samples were different, they diverged substantially more after 24 and 72 h of gonad culture (Fig. 5C). Differential gene expression analysis revealed that 211 and 3437 genes were increased, and 217 and 2160 genes were decreased in XY germ cells after 24 and 72 h compared to E12.5 (time 0) XY germ cells, respectively (Fig. 5B, Additional file 6: Table S1.3–1.4). Similarly, 340 and 3214 genes were increased, and 157 and 2592 genes were decreased in XX germ cells compared to E12.5 XX germ cells after 24 and 72 h (Fig. 5B, Additional file 6: Table S1.5–1.6). Together, these data indicated that while male and female germline differentiation progressed in the first 24 h, the greatest transcriptional change occurred between 24 (~ E13.5) and 72 h (~ E15.5).

To identify genes specifically associated with male and female germline differentiation, we compared XY with XX germ cells from DMSO controls. This revealed 589 and 3548 genes higher in XY than XX germ cells after 24 and 72 h (XY germline genes; Fig. 5B, Additional file 6: Table S1.7–1.8), including male germline genes *Dnmt3l, Dppa4, Nanos2, Piwil1, Piwil2, Piwil4, Tdrd1, Tdrd5* and *Tdrd9*. By comparison, 978 and 2898 genes were higher in XX than XY germ cells after 24 and 72 h (XX germline genes; Fig. 5B, Additional file 6: Table S1.9–1.10), including female germline and meiosis markers *Atr, Atm, Chek1, Ccnb3, H2ax, Irx3, Irx5, Msx1, Id1, Id2, Id3, Sycp2, Sycp3* and *Stra8*, confirming that E12.5 germ cells diverged in the expected sex-specific manner over time.

### FGF and MEK1/2 commonly regulate a subset of genes involved in germ cell tumours, stem cell biology and proliferation

To determine the initial impacts of MEKi and FGFRi on germline development, we compared outcomes in XY germ cells cultured with DMSO or 500 nM FGFRi or MEKi for 24 h. MDS indicated that germ cells from XX E12.5 + 24 h DMSO controls were transcriptionally distinct from all XY groups. XY E12.5 + 24 h DMSO, FGFRi or MEKi cultures were also transcriptionally distinct (Fig. 5D) and comparison of XY germ cells from MEKi and FGFRi cultures with DMSO controls revealed 43 and 25 DEGs, respectively (FDR < 0.05, |FC|≥ 1.5; Fig. 5E, Additional file 7: Table S2.1–2.2). These included 23 and 19 genes that were lower than expected (i.e. for which transcription was not properly activated) and 20 and six genes that were higher than expected (i.e. not properly repressed, or unexpectedly derepressed) in FGFRi or MEKi treatments. Nine genes were commonly dysregulated by MEKi and FGFRi implying that they depend on FGF signalling via MEK1/2 (Fig. 5Fi, Additional file 7: Table S2.3). Importantly, the direction of change (up- or downregulated) was concordant (roast test *P* = 0.00025) for each of the nine common DEGs, with eight expressed lower, and one higher than expected (Fig. 5Fii). The simplest interpretation is that the DEGs that were lower than expected were FGF responsive genes that depended on MEK1/2 signalling for their upregulation, and the DEGs that were higher than expected depended on FGF-MEK1/2 for their repression.

Further examination of the genes that were lower than expected revealed six FGFRi-MEKi DEGs associated with stem cell differentiation, cell self-renewal, cancer, cell proliferation and survival and germ cell tumours, including *Etv1* and *Etv4* [45–48], *Pdk1* [49], *Ret* [50, 51], *Tdgf1* [52] and *Calcoco2* [53]. Few genes commonly associated with sex-specific germline differentiation were represented in the FGFRi and MEKi DEG lists after 24 h of culture. However, although unaffected by FGFRi, *Nanos2*, which regulates male germline differentiation [54], was 9.12-fold lower than control in 24-h MEKi samples (Additional file 7: Table S2.2), consistent with a requirement for MEK1/2 signalling for its upregulation.

### Male-specific germline differentiation depends on MEK1/2, but FGF signalling is dispensable in E12.5 testes

To determine the impacts of FGFRi and MEKi on later stages of male germline differentiation, we analysed samples after 72 h of culture. Surprisingly, germ cells from 72 h XY DMSO and FGFRi cultures were transcriptionally similar (Fig. 5G). Although 43 genes were differentially expressed after 24 h of FGFR inhibition (FDR < 0.05, |FC|≥ 1.5; Fig. 5Ei, Additional file 7: Table S2.1), only one DEG was identified after 72 h and was higher than expected (FDR < 0.05, |FC|≥ 1.5; Fig. 5Hi, Additional file 7: Table S2.4). This may be because *Fgf9* transcription in the testis is normally diminished to very low levels by E14.5 [9]. In contrast, MEKi samples were transcriptionally distinct from XX and XY DMSO controls at 72 h (Fig. 5G), with 817 genes lower and 586 higher than controls (FDR < 0.05, |FC|≥ 1.5; Fig. 5Hii, Additional file 7: Table S2.5). Of the 817 genes that were lower, 686 were male germline genes (i.e. normally upregulated in the germ cells of XY vs XX E12.5 + 72 h DMSO cultures defined in Fig. 5B) and were therefore defined as MEK1/2-dependent male germline genes (Fig. 5Ii, Additional file 7: Table S2.6). Genes lower than expected included key male germline markers *Dppa4*, *Nanos2*, *Dnd1*, *Nxf2* and *Brdt* [54, 55]. Interestingly, the transcriptional levels of *Dppa4* and the germline teratoma gene *Dnd1* [56, 57] was similar to E12.5 XY germ cells (Fig. 5J), consistent with our observations of DPPA4 protein expression in MEKi-treated samples (Fig. 3A,B). In addition, DNA methylation and piRNA-associated genes, including *Dnmt3a*, *Dnmt3l*, *Tdrd1*, *Tdrd5*, *Tdrd9*, *Spocd1*, *Piwil1*, *Piwil4* and *Henmt1* [58–60], were not properly upregulated in MEKi-treated samples, with levels remaining substantially lower than in XY DMSO-treated controls (Fig. 5J). Consistent with persistent germ cell proliferation after MEK1/2 inhibition (Fig. 1Eii), genes regulating the G1-S transition, DNA synthesis and germ cell proliferation, including *Mki67, Pcna*, *Ccne1*, *Ccne2* and *Ccnd1*, remained high after 72 h of MEKi (Fig. 5J).

### MEK1/2 inhibition did not result in overt female germline differentiation

Of the 586 genes that were higher than control in XY germ cells after MEKi (i.e. genes derepressed or not properly repressed via MEK1/2 signalling), 382 were normally expressed at higher levels in differentiating XX than XY germ cells, suggesting feminisation of the germline (Fig. 5Iii; Additional file 7: Table S2.7). However, of these 382 genes, 218 were expressed at similar levels in E12.5 germ cells (Additional file 8: Fig. S6), consistent with MEKi blocking germline differentiation rather than increasing feminisation. These included genes marking germ cell proliferation such as *Ccne1, Pcna* and *Mki67* [2, 3] (Fig. 5J). As MEKi blocked mitotic arrest but most germ cells did not enter meiosis (Fig. 4C–F), the simplest explanation is that these genes remained high due to continued germ cell proliferation, rather than female differentiation.

MEKi upregulated *Stra8*, *Meioc*, *Irx3* and *Irx5* [3, 61–63] and some genes that normally increase during female germline development (genes in red box in Additional file 8: Fig. S6), but the female germline inducing gene *Bmp2* was not upregulated in somatic cells (Additional file 2: Fig. S2D). Moreover, genes marking meiosis initiation or progression including *Msx1, Sycp2, Sycp3*, *Ccnb3, Atr* and *Atm* [3, 64] were not significantly increased by MEKi (Fig. 5J). In addition, *Ccnb1*, which is normally repressed in differentiating XX germ cells [3], remained high in MEKi cultures (Fig. 5J). Other well-known meiosis markers including *Check1* and *H2ax* were transcribed higher in both MEKi and E12.5 XY controls than DMSO controls, so were not informative (Fig. 5J). Together, despite higher levels of some female germline markers including *Stra8* and *Meioc*, the low expression of many meiosis markers such as *Atr*, *Atm* and *Msx1* (Fig. 5J) was consistent with the limited entry of germ cells into meiosis indicated by analyses of cell cycle and p-gH2AX following MEK1/2 inhibition (Fig. 4C–F).

### MEK1/2 inhibition retained germ cells in a relatively undifferentiated state

MEK1/2 inhibition maintained XY germline proliferation and genes including *Dppa4, Dnd1, Ccne1, Ccne2, Mki67, Pcna, H2ax, Chek1* and *Ccnb1* remained at similar levels in E12.5 + 72 h MEKi samples as in E12.5 XY germ cells, indicating that MEKi blocked male germline differentiation (Fig. 5J). Consistent with this, the *Nodal* regulatory genes *Lefty1* and *Lefty2* were maintained at higher levels, although *Nodal* and *Tdgf1* were not affected (Fig. 5J). While *Nodal* and *Tdgf1* were not affected by MEKi at 72 h, the Nodal signalling target *Pitx2* also remained high in MEKi-treated samples (Fig. 5J).

Key regulators of pluripotency *Sox2* and *Nanog* are expressed in E12.5 XX and XY germ cells but are repressed after E13.5 [23, 55, 65]. MEKi maintained *Nanog*, but not *Sox2* transcription in germ cells (Fig. 5J). *Oct4* remained unchanged, but as *Oct4* transcription is maintained between E12.5 and E15.5 in XY germ cells [23], this was not informative (Fig. 5J). As expected, IF analysis revealed that E13.5 XY germ cells expressed OCT4 and SOX2 protein, but germ cells in XX DMSO + 72 h cultures were negative (Additional file 9: Fig. S7A). Although OCT4 and SOX2 staining intensity was significantly lower than in E13.5 XY gonads, the proportion of germ cells remaining positive and the staining intensity of these proteins was similar in XY gonads treated with DMSO, FGFRi or MEKi for 72 h (Additional file 9: Fig. S7B-E). In addition, 72 h MEKi altered genes associated with germ cell or other tumours [66, 67], or SSC function [68, 69] including higher *Dmrt1* and *Sox3* and lower *Rhox10* expression (Fig. 5J). Moreover, rather than being downregulated as normally occurs in differentiating male germ cells, *Etv3* was maintained at levels higher than or similar to E12.5 germ cells, but *Etv4* was repressed (Fig. 5J).

### MEK1/2 or FGF signalling inhibition in E12.5 XY gonads did not cause somatic sex reversal

Our experimental design was to use E12.5 gonad culture to examine germ cell fate within a gonadal environment in the absence of somatic sex reversal. While collecting germ cells, we also isolated the gonadal somatic cells from samples treated with DMSO, FGFRi and MEKi for 24 and 72 h. While the bulk of this data will be reported elsewhere, RNA-seq analysis revealed no change (based on FDR < 0.05, |FC|≥ 1.5) in the expression of *Foxl2, Rspo1, Bmp2* *Wnt4,* or *Fst *in samples treated with MEKi or FGFRi compared to DMSO controls (Additional file 2: Fig. S2D), consistent with IF analysis of SOX9, AMH and FOXL2 (Additional file 2: Fig. S2A-C). While there was no change in *Cyp26b1* in FGFRi-treated samples, MEKi significantly reduced *Cyp26b1* expression (Additional file 2: Fig. S2D), indicating that this gene may be regulated by MEK1/2, but not directly by FGF signalling. Together, with IF and flow analysis of SOX9, and IF staining of AMH and FOXL2 (Additional file 2: Fig. S2A-C), these data indicate that FGFRi or MEKi did not result in substantial somatic sex reversal of the gonads.

## Discussion

We have identified a novel and essential role for MEK1/2 signalling in male germ cell differentiation. Our data demonstrate that MEK1/2 signalling is required for XY germ cells to enter mitotic arrest, upregulate a wide range of genes that mark male germline differentiation and repress a range of female germline genes. This occurred in the apparent absence of somatic sex reversal as SOX9 and AMH expression were normal and ovarian markers were not upregulated. In contrast to MEK1/2, although FGF signalling has been implicated in directly promoting male germline differentiation, our data indicate that FGF signalling is dispensable from E12.5 for germ cell mitotic arrest and male germline differentiation. Instead, our data support a role for FGF-MEK1/2 signalling in regulating genes associated with Nodal signalling and stem cell characteristics in E12.5 XY germ cells, consistent with FGF and Nodal inducing pluripotency in XY germ cells and germ cell tumours, and the well-defined role for FGF in the derivation of pluripotent EGCs [9, 17, 41, 70–72].

MEK1/2 inhibition from E12.5 disrupted germ cell mitotic arrest, maintained expression of genes associated with germ cell proliferation and prevented appropriate upregulation of 686 male germline development genes. A prominent germline signature of MEKi was the deficient upregulation of de novo DNA methylation genes and piRNA pathway genes involved in silencing repetitive elements. This implies that the DNA methylation pathway either responds directly to MEK1/2 signalling or is not properly activated due to poor male germ cell differentiation when MEK1/2 is inhibited in the developing testis. Either way, germline DNA methylation is likely to remain low if MEK1/2 signalling is compromised in the germline, potentially allowing derepression of repetitive elements.

In addition, several genes associated with germline tumours, including members of the Nodal signalling pathway and genes associated with germline tumours in human GWAS and other studies, including *DMRT1* [67, 73], were expressed in germ cells of MEK1/2 inhibited samples. While we did not observe increased OCT4 or SOX2 protein levels, *Nanog* transcription remained high, germ cell proliferation was sustained, and germline differentiation was inhibited in the absence of MEK1/2 signalling. Combined with low DNA methylation, these factors could render germ cells more susceptible to germline tumours, a possibility that requires further investigation.

A range of studies demonstrate the ability of foetal germ cells to respond to FGF ligands, particularly in isolation from testicular somatic cells [15, 17, 21, 27]. Of particular note, FGF signalling (via FGF2, FGF5, FGF9 or FGF10) is required for inducing pluripotency during EGC derivation [17] and FGF9 induces MEK1/2-dependent proliferation in XY germ cells isolated from their somatic counterparts [21]. Moreover, FGF and MEK1/2 induce proliferation and underpin an undifferentiated state in SSCs. SSCs express the germline stem cell-associated genes *Etv5, Tdgf1, Ret* and, in a more dedifferentiated state, *Nanog* [19, 74]. Consistent with FGF promoting stem cell characteristics in foetal germ cells, we observed a requirement for FGF signalling to regulate 43 genes and MEK1/2 to regulate 25 genes in E12.5 germ cells after 24 h of culture. Nine genes were commonly dysregulated by FGFRi and MEKi, suggesting that these genes are regulated by FGF-MEK1/2-ERK1/2 signalling. Six of these genes, including *Etv1, Etv4, Ret, Tdgf1, Pdk1* and *Calcoco*, have been associated with germ cell tumours, stem cells, cell self-renewal, cancer, cell proliferation and cell survival [45–53]. Although we did not detect pERK1/2 in germ cells using IF, FGFR2 is expressed on the surface of germ cells [9] and it remains possible that pERK1/2 remained below detection levels and this response occurs directly in germ cells. Interestingly, *Nanos2* is also induced by FGF9 in SSCs and is required to maintain SSCs [19, 75]. *Nanos2* was not affected in 24 FGFRi cultures but was lower in 24 and 72 h MEKi cultures compared to DMSO control samples, demonstrating it was not properly upregulated when MEK1/2 was inhibited. While we did not detect pERK1/2 positive germ cells, our observations and those of others are consistent with FGF-MEK1/2 signalling priming stemness in germ cells.

Although inhibition of FGFR for 24 h from E12.5 resulted in dysregulation of 43 genes, only one gene was dysregulated after 72 h of FGFRi treatment, and germ cells entered mitotic arrest normally. In contrast, MEK1/2 inhibition in the same experiment dysregulated 1403 genes, precluded upregulation of the male germline markers DPPA4 and DNMT3L and prevented germ cells from entering mitotic arrest, demonstrating that MEKi disrupted male germline differentiation in E12.5 XY germ cells. Together, our data indicate that FGF signalling is dispensable, but MEK1/2 signalling in the developing testis is essential for normal differentiation of foetal male germ cells after E12.5.

It has been suggested that FGF9 directly induces male germline development [9, 15, 21]. However, while FGF9 resulted in higher *Nanos2* and *Dnmt3l* transcription in E11.5 XX and XY gonads, experiments in cultured foetal gonads and isolated germ cells have varied [9, 15, 20, 21]. In isolated E12.5 germ cells, 25 ng/ml FGF9 either did not significantly increase *Nanos2* or *Dnmt3l* transcription [9] or did increase *Nanos2* [15]. A third study found that 25 ng/ml FGF9 did not induce *Nanos2* or *Dnmt3l*, but 0.2 ng/ml FGF9 did [21]. Of note, ectopic FGF9 (20 ng/ml) or FGF2 (20 ng/ml) promoted SSC proliferation and these factors were able to induce self-renewal genes, including *Nanos2* in SSCs [19, 74]. Moreover, consistent with our observation that FGF9 was required for expression of stem cell-related genes in this study, FGF9 increased the pluripotency markers *Oct4* and *Sox2* in E12.5 germ cells [9, 20]. Together, it appears that while FGF9 is essential in the testis for promoting male somatic cell development and can induce stem cell characteristics in foetal germ cells, evidence that FGF9 directly promotes male germline development remains limited.

It has also been proposed that low levels of FGF9 (0.2 ng/ml) drive male germline differentiation, while high levels (25 ng/ml) promote stem cell characteristics [21]. We cannot exclude the possibility that residual FGF9 activity drives male germline development in the presence of FGFRi. However, several observations suggest that residual levels of FGF signalling are unlikely to explain our data. Firstly, male germline differentiation proceeded normally after deletion of *Fgf9* together with *Wnt4* [16]. In addition, in this study, FGFRi completely abrogated FGF9-induced somatic cell proliferation in XX gonads and reduced Sertoli cell proliferation to a similar extent in E12.5 gonads. Furthermore, three independent FGFR inhibitors reduced Sertoli cell proliferation to a similar extent but did not affect germ cell mitotic arrest, even when using 2500 nM FGFRi or 5000 nM SU5402, which was previously used to target FGF signalling [9]. Finally, p38MAPK or PI3K inhibition using two different inhibitors did not affect male germ cell mitotic arrest, indicating that residual FGF9 signalling via p38MAPK or PI3K is unlikely to explain why FGFRi failed to disrupt male germline development.

As FGF9 is at maximal levels at E11.5 in XY gonads and rapidly declines thereafter [9, 11], it remains possible that inhibition of FGFR at E12.5 may have been too late to disrupt male germline development. We were unable to test this possibility as inhibition of FGF signalling at E11.5 is expected to cause somatic sex reversal [6, 7, 12–14] and would substantially confound the study of sex-specific germ cell development. Nonetheless, MEK1/2 inhibition at E12.5 profoundly disrupted male germline development but FGFRi did not. It is possible that FGF9 acts on germ cells at E11.5 and then MEK1/2 signalling is required at E12.5. However, this would require either a temporal gap between FGFR activation and MEK1/2 signalling, or sustained FGF-MEK1/2 signalling to promote male germline development. Sustained FGF-MEK1/2 signalling would require both FGF and MEK1/2 signalling at E12.5, but our data indicate that only MEK1/2 is required. A temporal gap would require a lag of up to 12–24 h (i.e. between E11.5/12 and E12.5) between FGF and MEK1/2-pERK1/2 activation. However, FGF induction of MEK1/2 and pERK1/2 typically occurs within 10–15 min [25, 26], indicating that such a lag is unlikely. Based on past studies and this study, it seems more plausible that FGF9 is dispensable for male germline development in mice, a conclusion that is consistent with normal male germline development in mice lacking *Wnt4* and *Fgf9* [16].

**Fig. 6:**
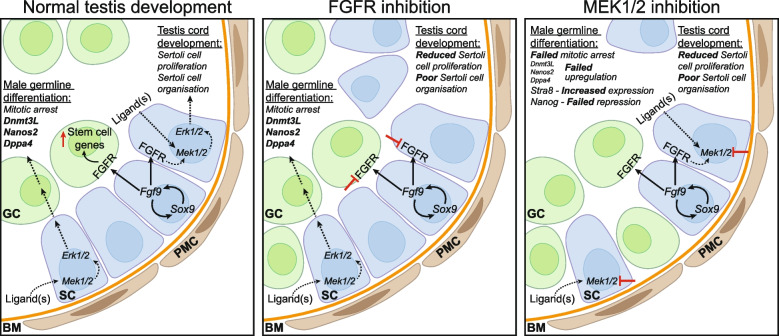
Proposed model for FGF and MEK1/2 signalling in testis and male germline development. In the developing testis, SOX9 and FGF9 promote Sertoli cell proliferation and organisation. It has been proposed that FGF9 drives both germ stem cell characteristics and male germline differentiation. Sertoli cells indirectly promote male germline differentiation, including mitotic arrest and expression of male germline markers such as *Nanos2*, *Dppa4* and *Dnmt3L.* MEK1/2 signalling inhibition results in failed germ cell mitotic arrest, failed upregulation of male germline markers including *Nanos2*, DPPA4 and DNMT3L and maintained expression of the pluripotency marker, *Nanog.* Although FGF signalling promotes Sertoli cell development and stem cell characteristics in foetal germ cells, it is dispensable for male germline differentiation. We propose that FGF-independent MEK1/2 signalling via an unknown ligand(s) also promotes Sertoli cell proliferation and organisation to indirectly facilitate male germline development

A model we favour is that FGF9 promotes Sertoli cell and testis development at E11.5 and an additional ligand(s) indirectly promotes male germline fate via MEK1/2 at E12.5 (Fig. 6). A combination of signalling by FGF and the other ligand(s) would explain the differing impacts of FGFRi and MEKi on both Sertoli cell proliferation and pERK1/2 in Sertoli cells in this study (Fig. 6). While FGFRi completely blocked FGF9-induced proliferation of XX somatic cells, FGFRi had a more modest effect on Sertoli cell proliferation than MEKi. Consistent with this, while MEKi completely abrogated pERK1/2 in Sertoli cells, FGFRi did not and FGFRi reduced Sertoli cell proliferation to a lesser extent than MEKi. It is tempting to speculate that PGD2 may be involved as it is known to facilitate *Sox9* induction and Sertoli cell proliferation and can activate MEK1/2-pERK1/2 in human keratinocytes [1, 8, 40]. Moreover, deletion of *L/H Ptgds* in mice enhanced E13.5 germ cell proliferation, allowed some germ cells to escape mitotic arrest and expression of proliferation and male germline differentiation genes was altered, while addition of PGD2 had opposing effects [1, 8, 40, 76]. However, other ligand(s) may also promote testis development and/or indirectly or directly drive male germline differentiation.

Deletion of FGF9 caused the loss of germ cells from E11.5, though some surviving germ cells entered meiosis [12]. In another study, while FGF9 treatment of isolated E11.5 germ cells demonstrated that FGF9 can reduce retinoic acid (RA) induced *Stra8* transcription, exposure of germ cells to FGF9 in the absence of RA did not significantly affect *Stra8* expression [9]. Moreover, FGFR inhibition in cultured E11.5 XX or XY gonads did not affect *Stra8* expression, but inhibition of CYP26B1 led to robust *Stra8* transcription, presumably due to greater RA availability [9]. This effect was enhanced by FGF9 inhibition in XY gonads but not XX gonads [9]. While this suggests that FGF9 and CYP26B1 act in concert to inhibit *Stra8* transcription, the effect of FGF9 in this context may also be due to an indirect effect on somatic cells and subsequent *Cyp26b1* transcription in E11.5 gonads rather than a direct effect on germ cells. In our study *Stra8* expression was not affected by FGFR inhibition in E12.5 gonads, perhaps because FGFRi did not reduce *Cyp26b1* transcription and CYP26B1 mediated RA degradation. In contrast, *Cyp26b1* was transcriptionally decreased in MEKi-treated E12.5 testes and *Stra8* transcription and protein were both increased. Despite this, in MEKi-treated samples STRA8 protein was largely localised to the germ cell cytoplasm and *Bmp2* expression was not increased in somatic cells, perhaps explaining why germ cells did not properly enter meiosis. Combined, it seems plausible that the primary role of FGF9 in *Stra8* repression is mediated by ensuring an appropriate testicular environment and consequent *Cyp26b1* expression and *Stra8* repression, rather than a direct effect of FGF9 on germ cells.

## Conclusions

Together our study reveals a novel, essential role of MEK1/2 signalling in foetal male germline differentiation, particularly in the promotion of germ cell mitotic arrest, the expression of an appropriate male germline transcriptional programme and the activation of de novo DNA methylation. While FGF9 may be involved in priming male germline development, it appears to be dispensable for male germ cell differentiation. Moreover, our data suggest that unknown ligand(s) activate MEK1/2 signalling and promote germline differentiation through an indirect mechanism (Fig. 6).

## Methods

### Mouse strains, animal housing, breeding and ethics

Mice were housed at Monash Medical Centre Animal Facility with controlled temperature and humidity, a 12 h light–dark cycle and food and water available ad libitum. Mouse embryos were obtained from inbred 129T2svJ *Oct4*-eGFP males crossed with Swiss females. Females were checked daily for vaginal plugs, with detection of a plug noted as E0.5. Animal work was undertaken in accordance with Monash Medical Centre Animal Facility Animal Ethics Committee approval.

### Organ culture

E12.5 and E13.5 embryos were sexed visually by the presence (male) or absence (female) of testis cords in the gonad. Gonad-mesonephros samples were cultured on 30-mm Millicell Biopore membranes with 0.4-μm pores (Merck Millipore; PICM03050) in 6-well plates, with each well containing 1400 μL media (15 mM Hepes, 0.1 mM non-essential amino acids, 1 mg/mL N-acetylcysteine, 1X penicillin/streptomycin, 55 μM beta-mercaptoethanol and 10% foetal calf serum in DMEM/F12 with Glutamax). PBS was placed in between the wells to maintain humidity. Gonads were cultured in media containing DMSO (vehicle control, used at a dilution of ≥ 1/5000, as appropriate for the concentration of each drug dilution), BGJ398 (FGFRi; SelleckChem, HY-13241), PD0325901 (MEKi; SelleckChem, S1036 or MedChem Express, HY-10254), GSK1059615 (PI3Ki; MedChem Express, HY-12036) and PH797804 (p38i; MedChem Express, HY-10403) using doses described in Table 1. Additional inhibitors for FGFR (AZD4547; SelleckChem, S2801 and SU5402; MedChem Express, HY-10407), p38 (Ralimetinib dimesylate; MedChem Express, HY-13241) and PI3K (PF-04691502; MedChem Express, HY-15177) signalling were also used (Table 1) to ensure consistency of outcomes with each of the primary inhibitors used. All inhibitors were selected based on their high specificity, potency and advancement in clinical trials (Table 1). Gonad-mesonephric complexes were randomly allocated to each culture treatment condition and cultured for 24, 48, 72 or 96 h in 5% CO_2_ at 37 °C, with media refreshed daily. To facilitate analysis of cell proliferation, EdU was added to each sample for the final 2 h of culture at a final concentration of 20 μM. After culture, gonads were photographed under bright-field and fluorescence optics, then processed for flow cytometry, IF or FACS. For gonad only flow cytometric experiments and all experiments involving FACS purification of germ and somatic cells, gonads were dissected away from mesonephros at the end of the culture period.

### Flow cytometry

Gonad collection, dissociation, fixation, staining and flow cytometry were performed essentially as described previously [77], using eGFP or antibodies specific for MVH, DPPA4, SOX9, DNMT3L, STRA8 or H2AX. Mesonephros or limb samples were used as germ cell negative controls to set gates for eGFP, MVH or DPPA4 and E12.5 female gonads were used as a negative control to set gates for SOX9 and DNMT3L. Cultured male gonads were used as a negative control to set gates for STRA8 and H2AX. Representative gating and negative control gates can be found in Additional file 10: Fig. S8. A rabbit IgG antibody was used as a negative control for determining staining intensities using specific rabbit antibodies. Primary antibodies used are listed in Table 2. Secondary antibodies used include Alexa Fluor Donkey anti Goat 488 (Thermo-Fisher, A11055), Alexa Fluor Donkey anti Goat 647 (Thermo-Fisher, A31573), Biotin Donkey anti Rabbit (Thermo-Fisher, A16027) and Biotin Donkey anti Goat (Thermo-Fisher, A16009). Cell cycle analysis was performed as previously described [77], with germ cells or Sertoli cells identified by their expression of MVH or SOX9, respectively. Cells were stained with 20 μg/mL of PI, enabling quantitation of cellular DNA content. Proliferation was measured by gating EdU-positive cells to identify cells in S-phase, while cells in G0/G1 or G2/M were respectively identified by DNA contents estimated as 2n or 4n in the EdU negative population compared to DMSO controls. All flow cytometry was performed on a BD FACS Canto II analyser (BD, Biosciences).

**Table 2:** Antibodies for flow cytometry (F) and immunofluorescence (IF)

Protein	Source and catalogue #	Species	Dilution
MVH	R&D Systems, AF2030	Goat	F: 1/100 IF: 1/400
DPPA4	R&D Systems, AF3730	Goat	F: 1/100 IF: 1/400
OCT4	Santa Cruz, sc8628	Goat	IF: 1/400
AMH	Santa Cruz, sc68886	Goat	IF: 1/200
MVH	Cell Signalling Technology, 8761S	Rabbit	IF: 1/400
SOX9	Sigma-Aldrich, AB5535	Rabbit	F: 1/200 IF: 1/1000
DNMT3L	Cell Signalling Technology, 13451S	Rabbit	F: 1/100 IF: 1/100
STRA8	Abcam, ab49405	Rabbit	F: 1/500 IF: 1/400
Phospho-γH2AX	Cell Signalling Technology, 9718S	Rabbit	F: 1/100 IF: 1/800
IgG	Cell Signalling Technology, 3900S	Rabbit	Equal to primary antibody used
Laminin	R&D Systems, L9393	Rabbit	IF: 1/200
PIWIL2	Cell Signalling Technology, 5940S	Rabbit	IF: 1/200
Phospho-ERK1/2	Cell Signalling Technology, 4370S	Rabbit	IF: 1/200
FOXL2	A gift from A/Prof Dagmar Wilhelm	Rabbit	IF: 1/500
SYCP3	Abcam, ab97672	Mouse	IF: 1/200
SMA	Sigma-Aldrich, A2547	Mouse	IF: 1/1000
NR2F2	R&D Systems, PP-H7147-00	Mouse	IF: 1/400
SOX2	Cell Signalling Technologies, 4900S	Mouse	IF: 1/100

### Tissue fixation, embedding, immunofluorescence and image analysis

Gonads were fixed in 4% paraformaldehyde (PFA) in PBS overnight at 4 °C. Samples were washed three times in PBS before 70% ethanol processing, and embedded in paraffin. Four-micrometre sections were cut in a compound series (typically four slides prepared per gonad sample with approximately 6 sections collected/slide), mounted on Superfrost Plus slides and dried at least overnight before antibody incubation. Antigen retrieval was conducted using Dako Citrate buffer (pH 6.0) for 30 min at 98 °C in a PT Link rinse station (Dako). Tissue sections were blocked in PBTx containing 5% BSA (Merck, A9647) and 10% donkey serum (Sigma; D9663) for 1 h at room temperature (RT). Primary antibody (Table 2) diluted in PBTx containing 1% BSA was left to incubate overnight at 4 °C or for 2 h RT. Slides were incubated for 1 h at RT in the dark in secondary antibody (Alexa Fluor, Thermo-Fisher, Donkey anti Goat 488 A11055 or Donkey anti Rabbit 488 A21206; Donkey anti Rabbit 647 A31573 or Donkey anti Mouse 647 A31571; Donkey anti Mouse 594 A21203; Donkey anti Goat 594 A11058 or Donkey anti Mouse 555 A31570) diluted at 1/300 in PBTx containing 1% BSA. Slides were mounted in ProLong Gold containing DAPI (Thermo-Fisher, P36931). Confocal images were taken using a Nikon C1 Confocal microscope, with images taken using either × 10 lens or × 40 oil immersion lens, or slides were scanned using a VS120 Virtual Slide microscope (Olympus), collecting single optical sections of the whole area for each section on the slide. Image analysis was conducted with QuPath (v0.3.0) [78].

### Statistical analysis

Flow cytometric data was analysed with FlowJo (v10.7.2) and GraphPad Prism (v9.2.0). Relative antibody staining intensities were calculated by removing background staining in negative control samples and normalising levels to the XY DMSO control set at 1.0. Data represents 3–16 biological replicates (outlined in figure legends and depicted in graphs). For IF analysis, data from at least three representative tissue sections from four biological replicates were averaged. Relative antibody staining intensity was calculated by normalising levels to the XY DMSO control set at 1.0. Data represents four biological replicates. Statistical significance was determined with GraphPad Prism (v9.2.0) using one-way ANOVA with Tukey’s multiple comparisons, two-way ANOVA with Tukey’s multiple comparisons or unpaired two-tailed *t*-test, where appropriate. If variance was unequal, a non-parametric Brown-Forsythe and Welch ANOVA with Dunnett’s T3 multiple comparisons was used. *P* values < 0.05 were considered significant. All error bars represent mean ± SEM. All experiments were replicated at least twice, with limited variation between experiments.

### Fluorescent activated cell sorting (FACS) of germ and somatic cells

After organ culture, the mesonephros was dissected from the gonads. Six to 15 gonads were pooled for each sample and were dissociated in trypsin. Germ and somatic cell populations were isolated as previously described [2, 77] using the BD FACSAria™ Fusion cell sorter. GFP-positive germ and GFP-negative somatic cell populations were isolated from E12.5 XX and XY gonads, E12.5 XX and XY control gonads cultured for 24 or 72 h in DMSO or XY E12.5 gonads cultured for 24 or 72 h in FGFRi or MEKi (*n* ≥ 4 for each treatment/group). Germ cells were defined as GFP positive, with dead PI-positive germ cells excluded.

### RNA sequencing library construction and sequencing

RNA was isolated from 3–45 × 10^4^ FACS-sorted germ cells using Macherey–Nagel NucleoSpin® RNA XS extraction kit (Scientifix, 740,902.50) following the manufacturer's instructions. RNA quantity and RNA integrity (RIN) were assessed using Qubit and Bioanalyzer (Agilent Technologies). Libraries were prepared with 30 ng of RNA from germ cells with RIN values greater than 7. The library was constructed by the MHTP Medical Genomics Facility as previously described [79]. Briefly, during initial poly(A) tail priming, an 8-bp sample index along with a 10-bp unique molecular identifier (UMI) was added. Samples were pooled and amplified with a template switching oligonucleotide. The Illumina P5 and P7 were added by PCR and Nextera transposase, respectively. The forward read (R1) utilises a custom primer to sequence into the index while the reverse read (R2) uses a standard R2 primer to sequence the cDNA in the sense direction. Seventeen indexes were added to enable parsing of sample sets. Sequencing was performed on NextSeq2000 (Illumina) following Illumina protocol 1,000,000,109,376 v3.

### Data preprocessing

FASTQ files were demultiplexed and mapped using scPipe [80] and Rsubread [81] (Bioconductor) packages in R studio. All code used for RNA sequencing analysis can be found in Additional file 11 and Additional file 12. Briefly, FASTQ files were reformatted with *sc_trim_barcode* to incorporate barcode information from read 1 into read 2 into the header. Reads were aligned to a reference mouse genome (GENECODE GRCm39) using Rsubread. Reads were assigned to annotated exons with sc*_exon_mapping*, data were demultiplexed using *sc_demultiplex* and a gene count matrix was generated with UMI deduplication using *sc_gene_counting*. Gene count matrices from each set were combined into a DGEList object for analysis.

### Downstream analysis of RNA sequencing data

Differential gene expression was assessed using the Limma [82], Glimma [83] and edgeR [84] Bioconductor packages following a previously established workflow [85]. Briefly, gene count data was loaded into R studio and genes were annotated with any duplicates removed. Raw counts were transformed into counts per million (CPM). Lowly expressed genes were removed using the *filterByExpr* function in edgeR, and gene expression distributions were normalised using trimmed mean of M-values (TMM) method [86]. MDS plots were generated to visualise sample clustering. Since there were many samples present, groups were subdivided into smaller groups to visualise clustering between different culture treatments and culture periods more clearly. Heteroscedasticity was removed from the count data with *voomWithQualityWeights* [87]. Linear modelling and empirical Bayes moderations was used to test for differential expression. As there were many DEGs identified following empirical Bayes moderations, to identify DEGs of biological relevance, a log_2_fold-change cut-off was set at greater/less than 0.585 (equivalent to a FC of 1.5) using *treat* [88]. Genes were considered differentially expressed if they met the logFC cut-off and had an FDR adjusted *p*-value less than 0.05. DEGs identified in XY E12.5 + 24 h FGFRi vs XY E12.5 + 24 h DMSO (24 h FGFRi DEGs) and XY E12.5 + 24 h MEKi vs XY E12.5 + 24 h DMSO (24 h MEKi DEGs) were compared, with genes commonly affected by FGFRi and MEKi assessed using *roast* [89]. Venn diagrams were generated using InteractiVenn [90] and Heatmaps were generated using ClustVis [91].

### Supplementary Information

**Additional file 1:** **Figure S1.** FGFR or MEK1/2 inhibition reduced Sertoli cell proliferation, but only MEK1/2 inhibition disrupted germ cell mitotic arrest. **A-F** Flow cytometric analysis of Sertoli (**A**,** C**,** E**) or germ (**B**,** D**,** F**) cell proliferation based on EdU incorporation in XY E12.5 gonad-mesonephros tissue cultured for 72 h with DMSO, 125, 250 or 500 nM of FGFR inhibitor, AZD4547 (**A**,**B**), 500 nM of p38 inhibitor, ralimetinib dimesylate or 500 nM of PI3K inhibitor, PF-04691502 (**C**,**D**) or 500 nM of MEKi, 2500 nM of FGFRi or 5000 nM of FGFR inhibitor, SU5402 (**E**,**F**). **G** Flow cytometric analysis of gonadal somatic cell proliferation identified by EdU incorporation in E12.5 XX gonads/mesonephros tissue cultured for 48 h with DMSO, FGF9 (50 ng/mL), 500 nM of FGFRi (i), MEKi (ii), p38i or PI3Ki (iii) and FGF9 + FGFRi (i), FGF9 + MEKi (ii), FGF9 + p38i or PI3Ki (iii). **H** Wide view immunofluorescent images of E12.5 gonad-mesonephros tissue cultured for 24 h with DMSO, 500 nM of FGFRi or MEKi demonstrating MEK1/2 signalling activity. Top panel: DAPI (blue), MVH (green), NR2F2 (red), pERK1/2 (cyan). Bottom panel: pERK1/2 (grey). Scalebar represents 100 μm. **I** Flow cytometric analysis of Sertoli cell proliferation based on EdU incorporation in XY E12.5 gonad-mesonephros tissue cultured for 72 h with DMSO, 500 nM FGFRi or MEKi. Replicates: **A-D** *n *= 4, **E**,**F** *n *= 3-4, **Gi** *n *= 5-6, **Gii** *n *= 8-12, **Giii** *n *= 11-21. Statistics: **A-F**,** Gii**,** Giii** Ordinary one-way ANOVA with Tukey’s multiple comparison, **Gi**,**I** Brown-Forsythe and Welch ANOVA with Dunnett’s T3 multiple comparisons. Error bars: Mean ± SEM. Significance between controls and treatment: *P<0.05, **P<0.01, ***P<0.001, ****P<0.0001.

**Additional file 2:** **Figure S2.** FGF and MEK1/2 inhibition from E12.5 does not cause sex reversal of the gonads. Analysis of XY E12.5 or XX E12.5 gonad-mesonephros tissue cultured with DMSO or 500 nM of FGFRi or MEKi for 72 h. **A** Immunofluorescent images demonstrating AMH and SOX9 staining. Top panel: DAPI (blue), AMH (green), SMA (red), SOX9 (cyan). Middle panel: AMH (grey). Bottom panel: SOX9 (grey). **B** SOX9 staining intensity in Sertoli cells determined by flow cytometry. **C** Immunofluorescent images demonstrating FOXL2 staining. Top panel: DAPI (blue), MVH (green), NR2F2 (red), FOXL2 (cyan). Bottom panel: FOXL2 (grey). **D** RNA sequencing results in isolated gonadal somatic cells following 72 h culture of key female and male gonadal somatic cell markers including *Foxl2*,* Rspo1*,* Bmp2*,* Wnt4*, *Fst* and *Cyp26b1. *Data shows the fold-change between XX E12.5 DMSO v XY E12.5 DMSO, XY E12.5 FGFRi v XY E12.5 DMSO and XY E12.5 MEKi v XY E12.5 DMSO. Plus (+) or minus (–) symbol indicates increased or decreased expression, respectively. Asterisks indicates statistical significance based on FDR<0.05 and FC >1.5. Scale bar represents 100 μm. Replicates: **A**,**C** *n *= 3-4, **Bi** *n *= 8-11, **Bii** *n* = 3-16, **D** *n *= 4 Statistics: **Bi** Welch ANOVA with Dunnett’s T3 multiple comparisons, **Bii** Ordinary one-way ANOVA with Tukey’s multiple comparison. In **B**; Intensity is relative to DMSO control sample set at 1.0. Error bars: Mean ± SEM. Significance between controls and treatment: *P<0.05, ***P<0.001.

**Additional file 3:** **Figure S3.** Flow cytometric scatterplot depicting Sertoli and germ cell proliferation. Flow cytometric scatterplots of XY E12.5 gonad-mesonephros tissue cultured in DMSO, 125, 250, 500 or 1000 nM of FGFRi or MEKi for 72 h showing the percentage EdU incorporation in Sertoli (**A**) or germ (**B**) cells. Percentage in top left corner of each graph represents the average proportion of Sertoli (**A**) or germ (**B**) cells in each treatment.

**Additional file 4:** **Figure S4.** Widefield view of images displayed in Fig. 3. Immunofluorescent images of XY E12.5 gonad-mesonephros or XY or XX E12.5 gonad-mesonephros tissue cultured with DMSO or 500 nM of FGFRi or MEKi for 72 h. **A** Immunofluorescent images demonstrating DPPA4 localisation. Top panel: DAPI (blue), MVH (green), DPPA4 (red) and SMA (cyan). Bottom panel: DPPA4 (grey). **B** Immunofluorescent images demonstrating DNMT3L localisation. Left panel: DAPI (blue), MVH (green), NR2F2 (red) and DNMT3L (cyan). Right panel: DNMT3L (grey). **C** Immunofluorescent images demonstrating PIWIL2 localisation Left panel: DAPI (blue), MVH (green), SMA (red) and PIWIL2 (cyan). Right panel: PIWIL2 (grey). Scale bar: 100 μm. Replicates: *n *= 3-4.

**Additional file 5:** **Figure S5.** Widefield view of images displayed in Fig. 4. Immunofluorescent images of XY or XX E12.5 gonad-mesonephros tissue cultured with DMSO, FGFRi or MEKi for 72 h (**A**,**B**) or 96h (**C**). **A** Immunofluorescent images demonstrating STRA8 localisation. Top panel: DAPI (blue), MVH (green), STRA8 (red) and SMA (cyan). Bottom panel: Stra8 (grey). **B**,**C** Immunofluorescent images demonstrating SCP3 and phospho-γH2AX (p-γH2AX) localisation. Left panel: DAPI (blue), MVH (green), SCP3 (red; **B**) or SMA (red; **C**) and phospho-γH2AX (cyan). Middle panel: SCP3 (grey; **B**). Right panel: p-γH2AX (grey). Replicates: *n *= 3-4. Scale bar: 100 μm.

**Additional file 6: Table S1.** Supplementary Table containing gene lists generated from RNA sequencing analyses performed in this study.

**Additional file 7: Table S2.** Supplementary Table containing gene lists generated from RNA sequencing analyses performed in this study.

**Additional file 8:** **Figure S6.** Heatmap of 382 72 h MEK1/2 dependent genes expressed higher than expected common in 72 h XX germline specific genes. Genes which were expressed higher than expected in XY E12.5 + 72h MEKi vs XY E12.5 + 72h DMSO and were present in the 72 h XX germline specific genes dataset (identified as genes which were upregulated in XX E12.5 + 72h DMSO vs XY E12.5 + 72h DMSO) were assessed. Of these 382 genes, 218 genes were highly expressed in XY E12.5 germ cells and XX E12.5 + 72h DMSO germ cells and were therefore not considered informative. 164 genes were not or were lowly expressed in XY E12.5 germ cells compared to XX E12.5 + 72h DMSO germ cells and were therefore considered more reliable female germline differentiation genes (identified by red box). Genes with an FDR <0.05 and |logFC| >0.585 (equivalent to |FC| >1.5) were considered differentially expressed.

**Additional file 9:** **Figure S7.** FGF and MEK1/2 inhibition does not result in abnormal maintenance of pluripotency markers. Immunofluorescent analysis of XY E13.5 gonad-mesonephros tissue or XY or XX E12.5 gonad-mesonephros tissue cultured with DMSO or 500 nM of FGFRi or MEKi for 72 h. **A** Whole view immunofluorescent images demonstrating OCT4 and SOX2 localisation. Left panel: DAPI (blue), MVH (green), OCT4 (red) and SOX2 (cyan). Middle panel: OCT4 (white). Right panel: SOX2 (white). Scale bar: 100 μm. **B**,**C** Percentage of OCT4+ (**B**) or SOX2+ (**C**) germ cells calculated from immunofluorescent images. **D**,**E** OCT4 (**D**) or SOX2 (**E**) intensity in germ cells relative to XY DMSO control set at 1.0, calculated from immunofluorescent images. Replicates: *n *= 4. Statistics: Brown-Forsythe and Welch ANOVA with Dunnett’s T3 multiple comparisons. Error bars: mean ± SEM. Significance between controls and treatment: *P<0.05, **P<0.01, ***P<0.001, ****P<0.0001.

**Additional file 10:** **Figure S8.** Representative plots depicting gating for antibodies used in flow cytometric analysis. A = Area, W = Width. **A** Gates used to separate cells from debris (left) and to isolate single cells based on propidium iodide staining (right). **B** Germ and somatic cells populations were identified by detecting *Oct4*eGFP transgene (left). E12.5 mouse limb or mesonephros cells were used as a negative control (right). **C** Germ and somatic cells populations were identified by detecting MVH staining (left). E12.5 mouse limb or mesonephros were used as a negative control (right). **D** Sertoli cells were identified based on SOX9 staining (left). XX somatic cells were used as a negative control (right). **E**,**F** Incorporation of EdU was used to identify proliferating Sertoli cells (**E**) or germ cells (**F**), with PI incorporation used to determine individual cell DNA content (left). E12.5 limb or mesonephros (**E**) or E12.5 XX germ cells not exposed to EdU (**F**) were used as a negative control for EdU (right). **G** DNMT3L+ germ cells identified with DNMT3L staining (left). XX germ cells were used as a negative control (right). **H** DPPA4+ germ cells were identified with DPPA4 staining (left). Cells not stained for DPPA4 were used as a negative control (right). **I**,**J** E12.5 + 72h XY DMSO germ cells were used as a negative control for STRA8 (**I**) or p-γH2AX (**J**) staining (left). E12.5 + 72h XX DMSO germ cells were used as a positive control (right).

**Additional file 11.** R code for RNA sequencing. All code used for RNA sequencing analysis. Please note that the RNA sequencing data set contained both germ cell and somatic cell data, the latter of which will be published elsewhere. Only the code relating to this study of germ cells has been provided.

**Additional file 12.** design.txt. File containing the labels for RNA sequencing data to be applied to the DGE list.

**Additional file 13.** Individual data values. Individual data values for experiments with n < 6.

## Data Availability

All data generated or analysed during this study are included in this published article, its supplementary information files and publicly available repositories. The RNA sequencing data have been deposited in the Gene Expression Omnibus (GEO) and are publicly available via accession number GSE221453 [Blucher RO, Lim RS, Jarred EG, Ritchie ME, Western PS. FGF-independent MEK1/2 signalling is essential for male germline development in mice (Germ Cell), and GSE221458 [Blucher RO, Lim RS, Jarred EG, Ritchie ME and Western PS. FGF-independent MEK1/2 signalling is essential for male germline development in mice (Somatic Cell)]. The individual data values for all data with n  < 6 (Figs. 1Dii, Eii, F, G, 3B, D, E, Additional file 1: Fig. S1A-F, Gi, Additional file 2: Fig. S2Bii, and Additional file 9: Fig. S7B-E) are provided in Additional file 13.

## Abbreviations

CPM: Counts per million
DEG: Differentially expressed gene
DMSO: Dimethyl sulfoxide/vehicle control
Dnmt3l: DNA methyltransferase 3 like
E: Embryonic day
EdU: 5-Ethynyl-2’-deoxyuridine
EGC: Embryonic germ cell
ERK1/2: Extracellular signal-regulated kinase 1/2
ETS: E26 transformation-specific
FACS: Fluorescent activated cell sorting
FC: Fold-change
FDR: False discovery rate
FGF: Fibroblast growth factor
FGF9: Fibroblast growth factor 9
FGFR: FGF receptor
FGFRi: FGFR inhibitor
IF: Immunofluorescence/immunofluorescent
MAPK: Mitogen-activated protein kinase
MDS: Multidimensional scaling
MEKi: MEK1/2 inhibitor
MVH: Mouse vasa homolog
Nanos2: Nanos C2HC-type zinc finger 2
p-γH2AX: Phosphorylated γH2AX
p38i: P38MAPK inhibitor
pERK1/2: Phosphorylated ERK1/2
PFA: 4% Paraformaldehyde
PGC: Primordial germ cell
PGD2: Prostaglandin D_2_
PI: Propidium iodide
PI3Ki: PI3K inhibitor
R1: Forward read
R2: Reverse read
RA: Retinoic acid
RIN: RNA integrity
RT: Room temperature
SMA: Smooth muscle actin
Sox9: SRY box 9
Sry: Sex determining region Y
SSC: Spermatogonial stem cell
Stra8: Stimulated by retinoic acid gene 8
SYCP3: Synaptonemal complex protein 3
TMM: Trimmed mean of M-values
UMI: Unique molecular identifier
