# Supplementary figures and images for: FGF-independent MEK1/2 signalling in the developing foetal testis is essential for male germline differentiation in mice
Source: BMC Biol. 2023 Dec 5;21:281. doi: 10.1186/s12915-023-01777-x (PMC10696798; doi:10.1186/s12915-023-01777-x)

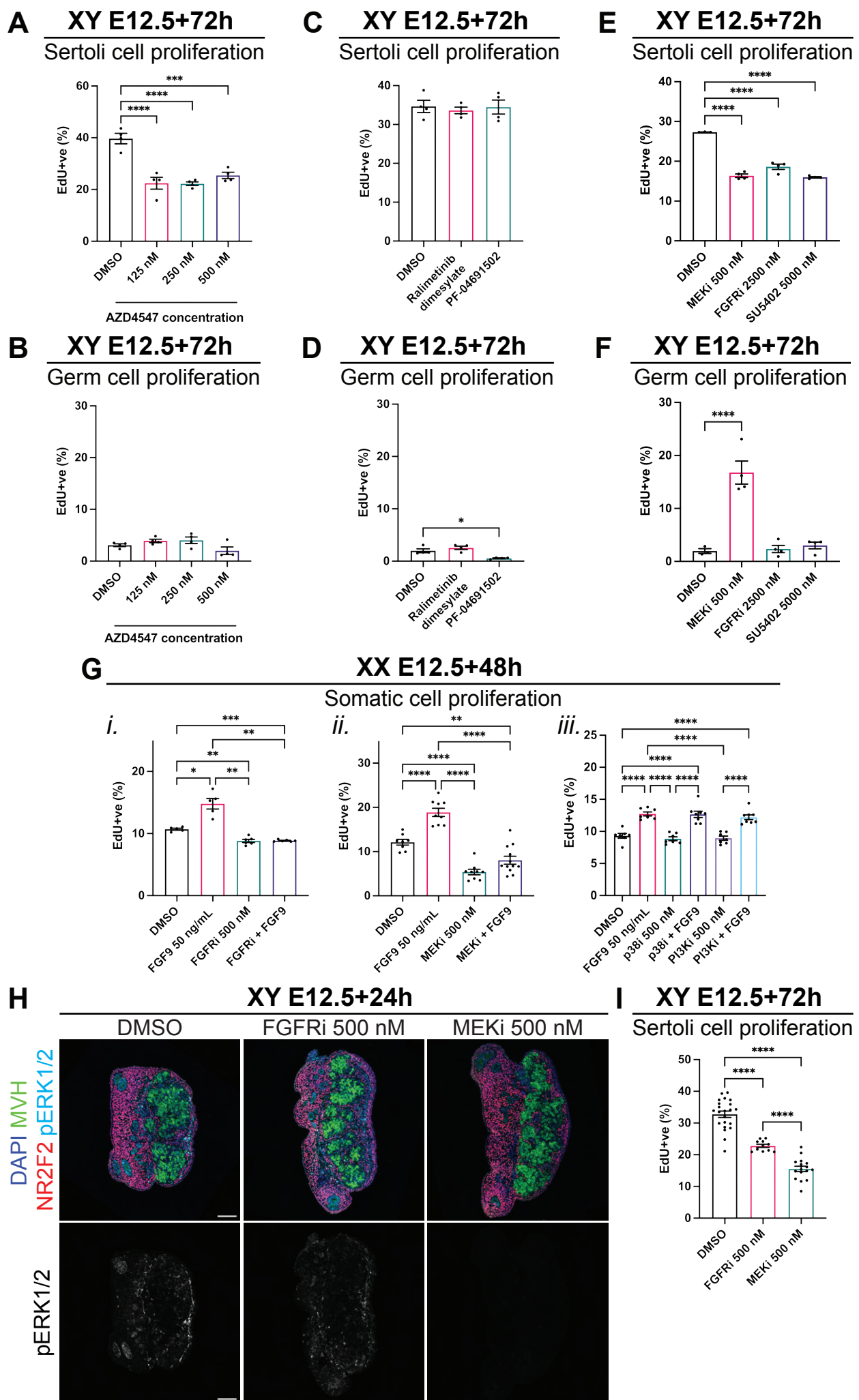

Supplement: Supplementary file 1 — Additional file 1: Figure S1. FGFR or MEK1/2 inhibition reduced Sertoli cell proliferation, but only MEK1/2 inhibition disrupted germ cell mitotic arrest. A-F Flow cytometric analysis of Sertoli (A, C, E) or germ (B, D, F) cell proliferation based on EdU incorporation in XY E12.5 gonad-mesonephros tissue cultured for 72 h with DMSO, 125, 250 or 500 nM of FGFR inhibitor, AZD4547 (A,B), 500 nM of p38 inhibitor, ralimetinib dimesylate or 500 nM of PI3K inhibitor, PF-04691502 (C,D) or 500 nM of MEKi, 2500 nM of FGFRi or 5000 nM of FGFR inhibitor, SU5402 (E,F). G Flow cytometric analysis of gonadal somatic cell proliferation identified by EdU incorporation in E12.5 XX gonads/mesonephros tissue cultured for 48 h with DMSO, FGF9 (50 ng/mL), 500 nM of FGFRi (i), MEKi (ii), p38i or PI3Ki (iii) and FGF9 + FGFRi (i), FGF9 + MEKi (ii), FGF9 + p38i or PI3Ki (iii). H Wide view immunofluorescent images of E12.5 gonad-mesonephros tissue cultured for 24 h with DMSO, 500 nM of FGFRi or MEKi demonstrating MEK1/2 signalling activity. Top panel: DAPI (blue), MVH (green), NR2F2 (red), pERK1/2 (cyan). Bottom panel: pERK1/2 (grey). Scalebar represents 100 μm. I Flow cytometric analysis of Sertoli cell proliferation based on EdU incorporation in XY E12.5 gonad-mesonephros tissue cultured for 72 h with DMSO, 500 nM FGFRi or MEKi. Replicates: A-D n = 4, E,F n = 3-4, Gi n = 5-6, Gii n = 8-12, Giii n = 11-21. Statistics: A-F, Gii, Giii Ordinary one-way ANOVA with Tukey’s multiple comparison, Gi,I Brown-Forsythe and Welch ANOVA with Dunnett’s T3 multiple comparisons. Error bars: Mean ± SEM. Significance between controls and treatment: *P<0.05, **P<0.01, ***P<0.001, ****P<0.0001. [file 12915_2023_1777_MOESM1_ESM.pdf]

**A**

# Sertoli cell proliferation

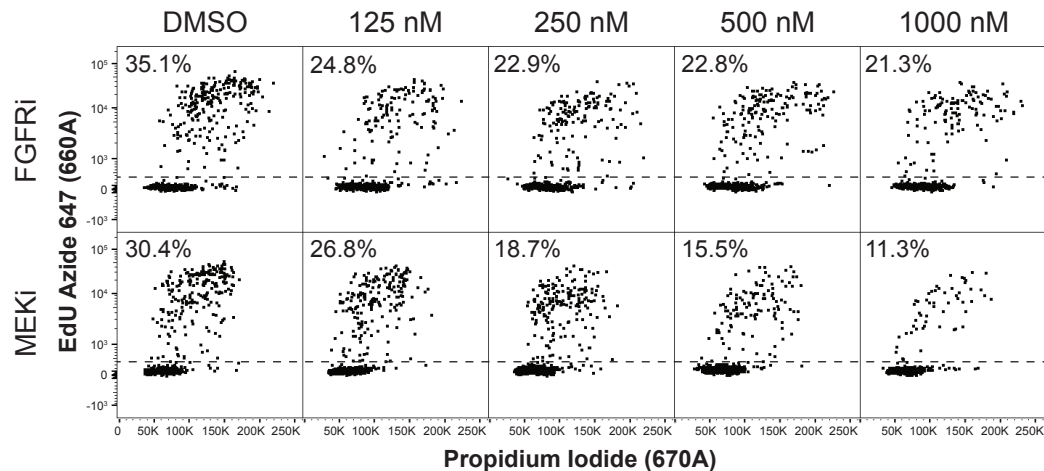**B**

# Germ cell proliferation

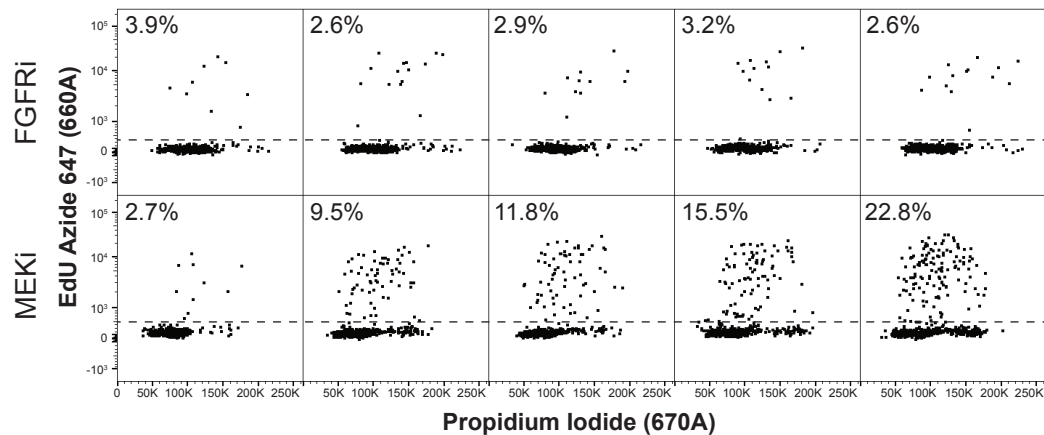

Supplement: Supplementary file 3 — Additional file 3: Figure S3. Flow cytometric scatterplot depicting Sertoli and germ cell proliferation. Flow cytometric scatterplots of XY E12.5 gonad-mesonephros tissue cultured in DMSO, 125, 250, 500 or 1000 nM of FGFRi or MEKi for 72 h showing the percentage EdU incorporation in Sertoli (A) or germ (B) cells. Percentage in top left corner of each graph represents the average proportion of Sertoli (A) or germ (B) cells in each treatment. [file 12915_2023_1777_MOESM3_ESM.pdf]

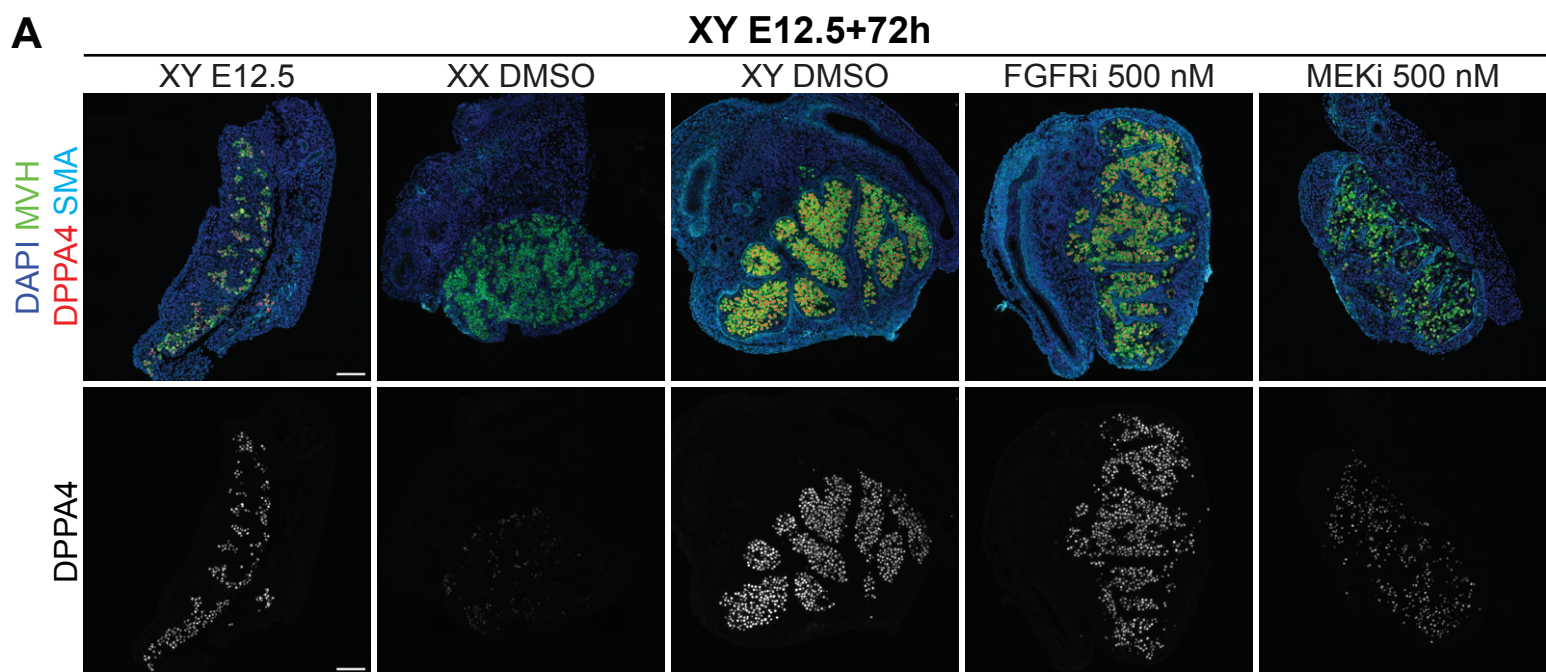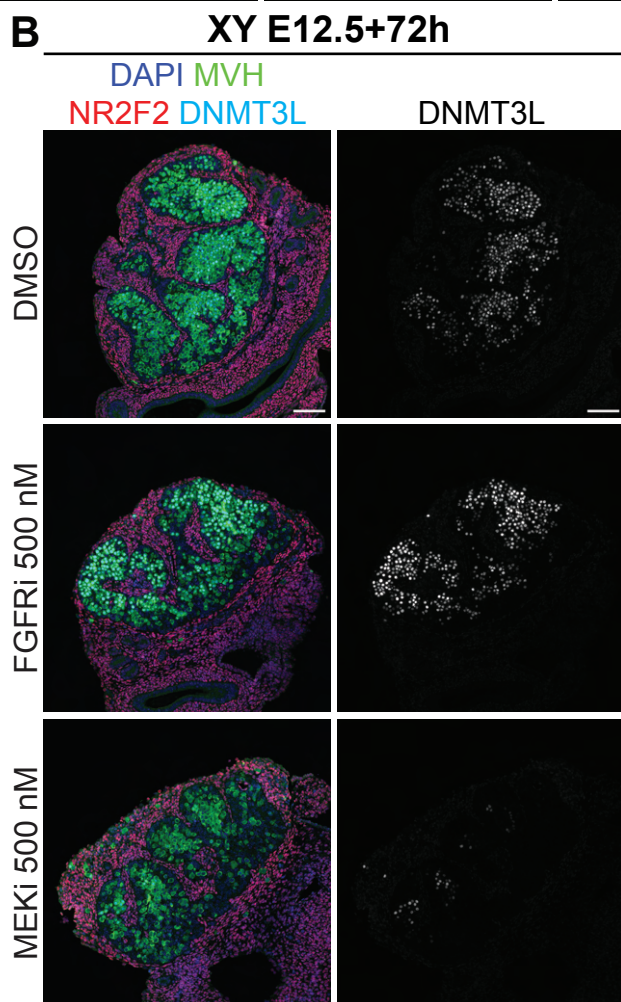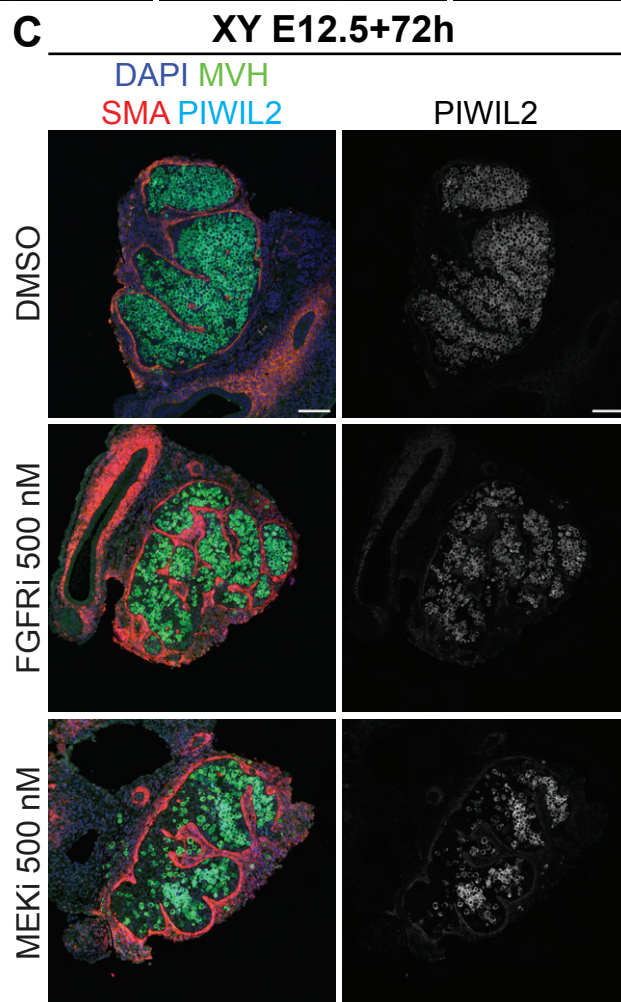

Supplement: Supplementary file 4 — Additional file 4: Figure S4. Widefield view of images displayed in Fig. 3. Immunofluorescent images of XY E12.5 gonad-mesonephros or XY or XX E12.5 gonad-mesonephros tissue cultured with DMSO or 500 nM of FGFRi or MEKi for 72 h. A Immunofluorescent images demonstrating DPPA4 localisation. Top panel: DAPI (blue), MVH (green), DPPA4 (red) and SMA (cyan). Bottom panel: DPPA4 (grey). B Immunofluorescent images demonstrating DNMT3L localisation. Left panel: DAPI (blue), MVH (green), NR2F2 (red) and DNMT3L (cyan). Right panel: DNMT3L (grey). C Immunofluorescent images demonstrating PIWIL2 localisation Left panel: DAPI (blue), MVH (green), SMA (red) and PIWIL2 (cyan). Right panel: PIWIL2 (grey). Scale bar: 100 μm. Replicates: n = 3-4. [file 12915_2023_1777_MOESM4_ESM.pdf]

**A****E12.5+72h**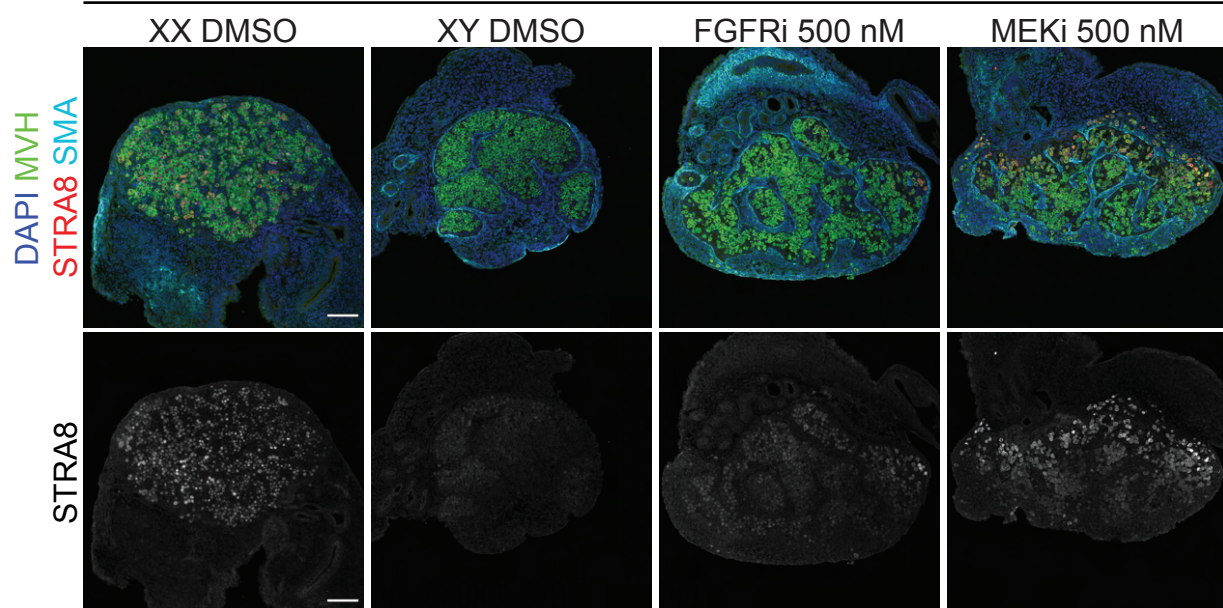**B****E12.5+72h**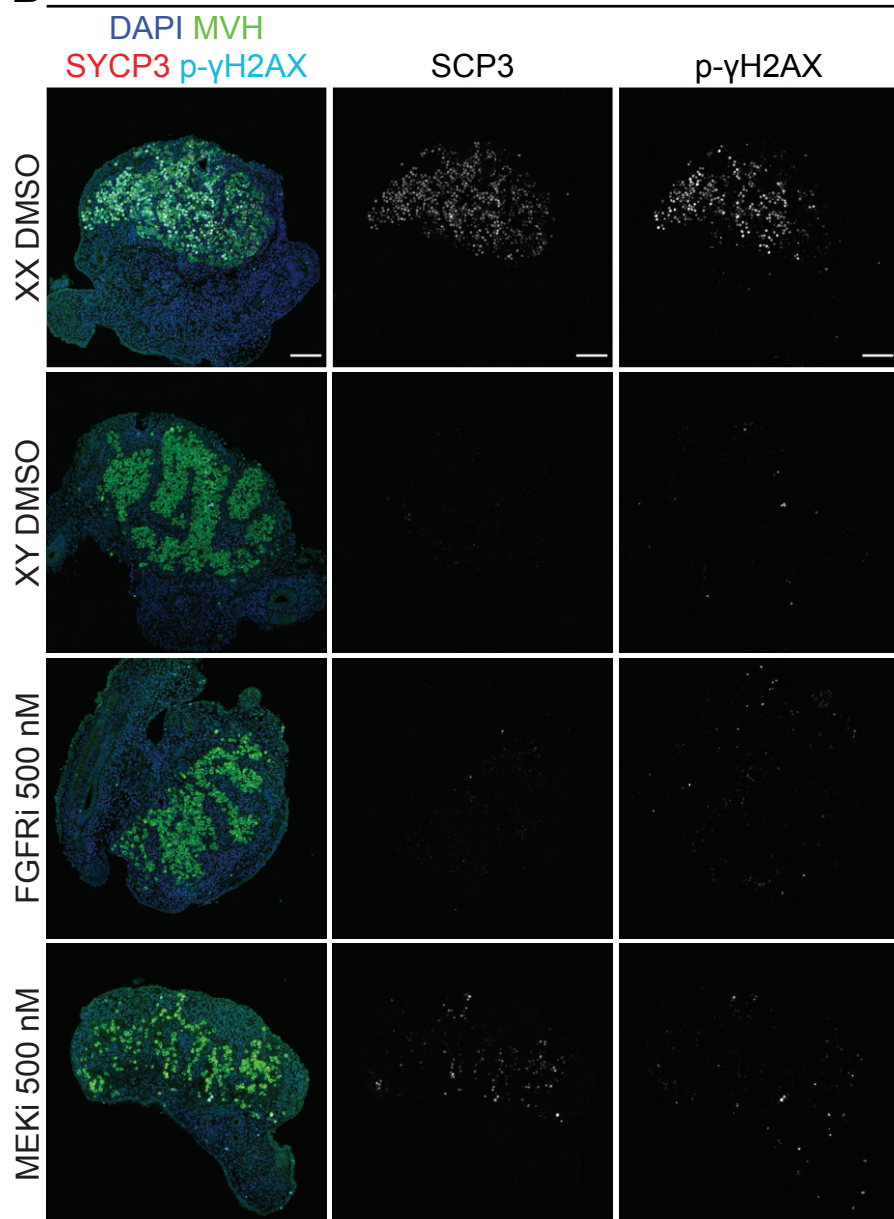**C****E12.5+96h**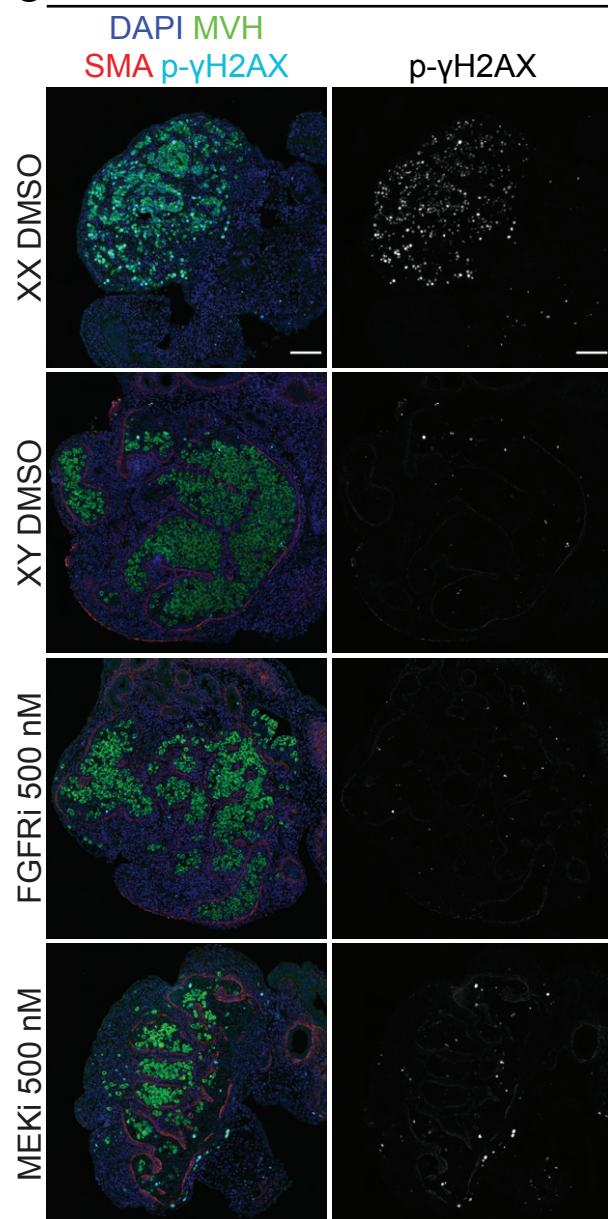

Supplement: Supplementary file 5 — Additional file 5: Figure S5. Widefield view of images displayed in Fig. 4. Immunofluorescent images of XY or XX E12.5 gonad-mesonephros tissue cultured with DMSO, FGFRi or MEKi for 72 h (A,B) or 96h (C). A Immunofluorescent images demonstrating STRA8 localisation. Top panel: DAPI (blue), MVH (green), STRA8 (red) and SMA (cyan). Bottom panel: Stra8 (grey). B,C Immunofluorescent images demonstrating SCP3 and phospho-γH2AX (p-γH2AX) localisation. Left panel: DAPI (blue), MVH (green), SCP3 (red; B) or SMA (red; C) and phospho-γH2AX (cyan). Middle panel: SCP3 (grey; B). Right panel: p-γH2AX (grey). Replicates: n = 3-4. Scale bar: 100 μm. [file 12915_2023_1777_MOESM5_ESM.pdf]

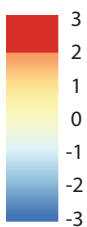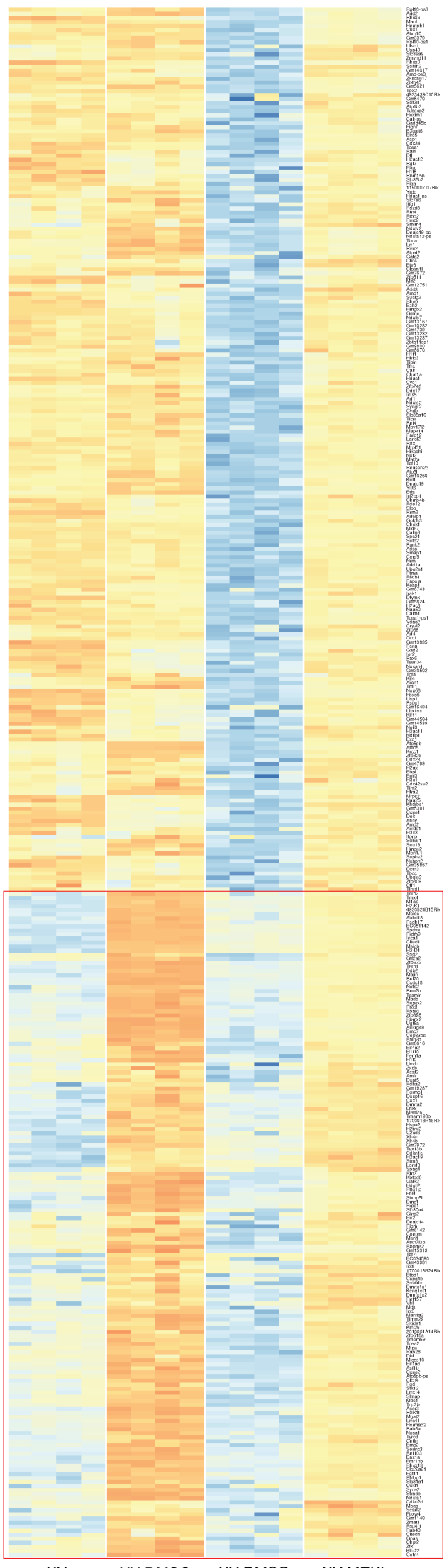

XY E12.5      XX DMSO 72h      XY DMSO 72h      XY MEKi 72h

Supplement: Supplementary file 8 — Additional file 8: Figure S6. Heatmap of 382 72 h MEK1/2 dependent genes expressed higher than expected common in 72 h XX germline specific genes. Genes which were expressed higher than expected in XY E12.5 + 72h MEKi vs XY E12.5 + 72h DMSO and were present in the 72 h XX germline specific genes dataset (identified as genes which were upregulated in XX E12.5 + 72h DMSO vs XY E12.5 + 72h DMSO) were assessed. Of these 382 genes, 218 genes were highly expressed in XY E12.5 germ cells and XX E12.5 + 72h DMSO germ cells and were therefore not considered informative. 164 genes were not or were lowly expressed in XY E12.5 germ cells compared to XX E12.5 + 72h DMSO germ cells and were therefore considered more reliable female germline differentiation genes (identified by red box). Genes with an FDR <0.05 and |logFC| >0.585 (equivalent to |FC| >1.5) were considered differentially expressed. [file 12915_2023_1777_MOESM8_ESM.pdf]

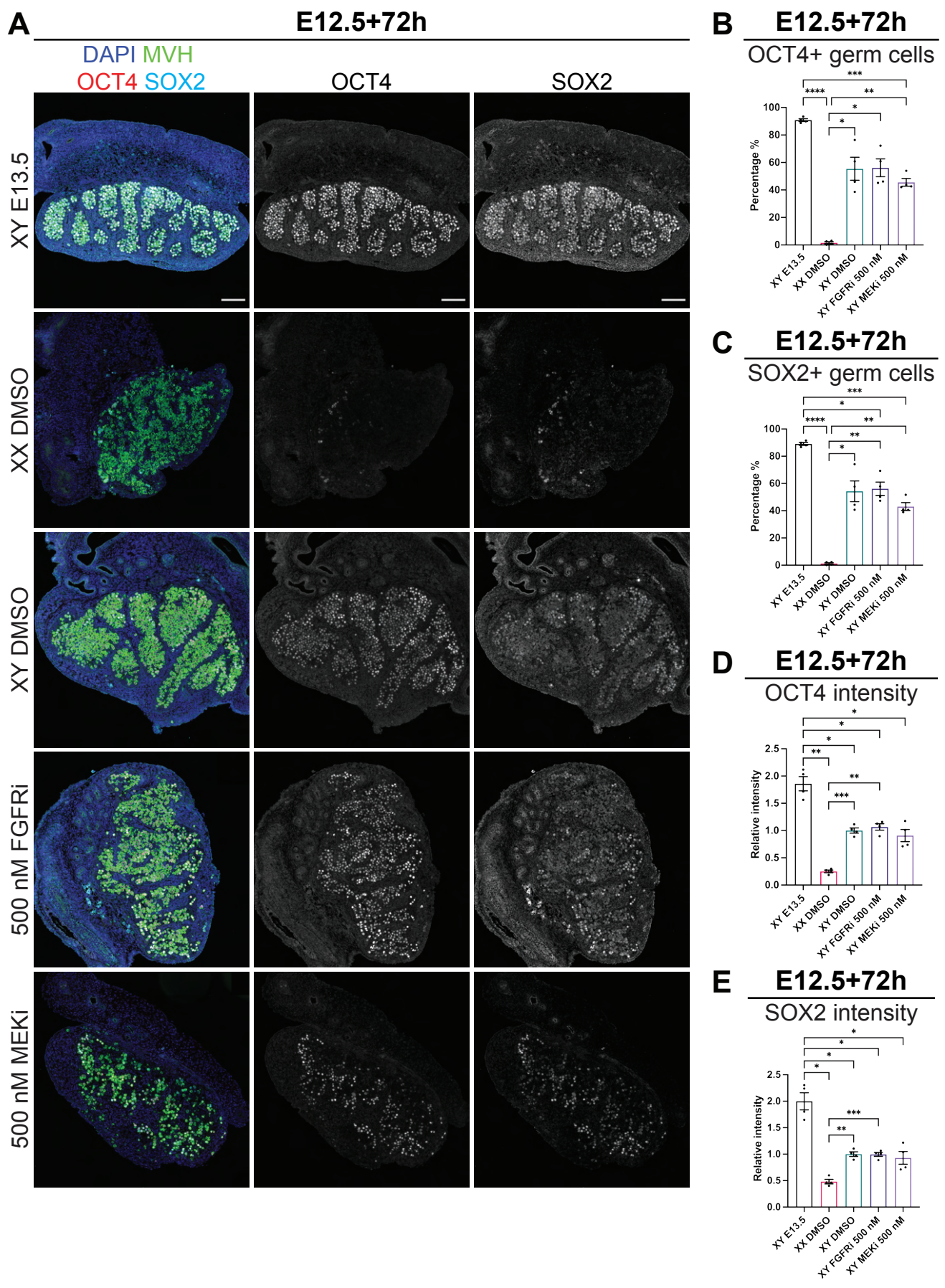

Supplement: Supplementary file 9 — Additional file 9: Figure S7. FGF and MEK1/2 inhibition does not result in abnormal maintenance of pluripotency markers. Immunofluorescent analysis of XY E13.5 gonad-mesonephros tissue or XY or XX E12.5 gonad-mesonephros tissue cultured with DMSO or 500 nM of FGFRi or MEKi for 72 h. A Whole view immunofluorescent images demonstrating OCT4 and SOX2 localisation. Left panel: DAPI (blue), MVH (green), OCT4 (red) and SOX2 (cyan). Middle panel: OCT4 (white). Right panel: SOX2 (white). Scale bar: 100 μm. B,C Percentage of OCT4+ (B) or SOX2+ (C) germ cells calculated from immunofluorescent images. D,E OCT4 (D) or SOX2 (E) intensity in germ cells relative to XY DMSO control set at 1.0, calculated from immunofluorescent images. Replicates: n = 4. Statistics: Brown-Forsythe and Welch ANOVA with Dunnett’s T3 multiple comparisons. Error bars: mean ± SEM. Significance between controls and treatment: *P<0.05, **P<0.01, ***P<0.001, ****P<0.0001. [file 12915_2023_1777_MOESM9_ESM.pdf]

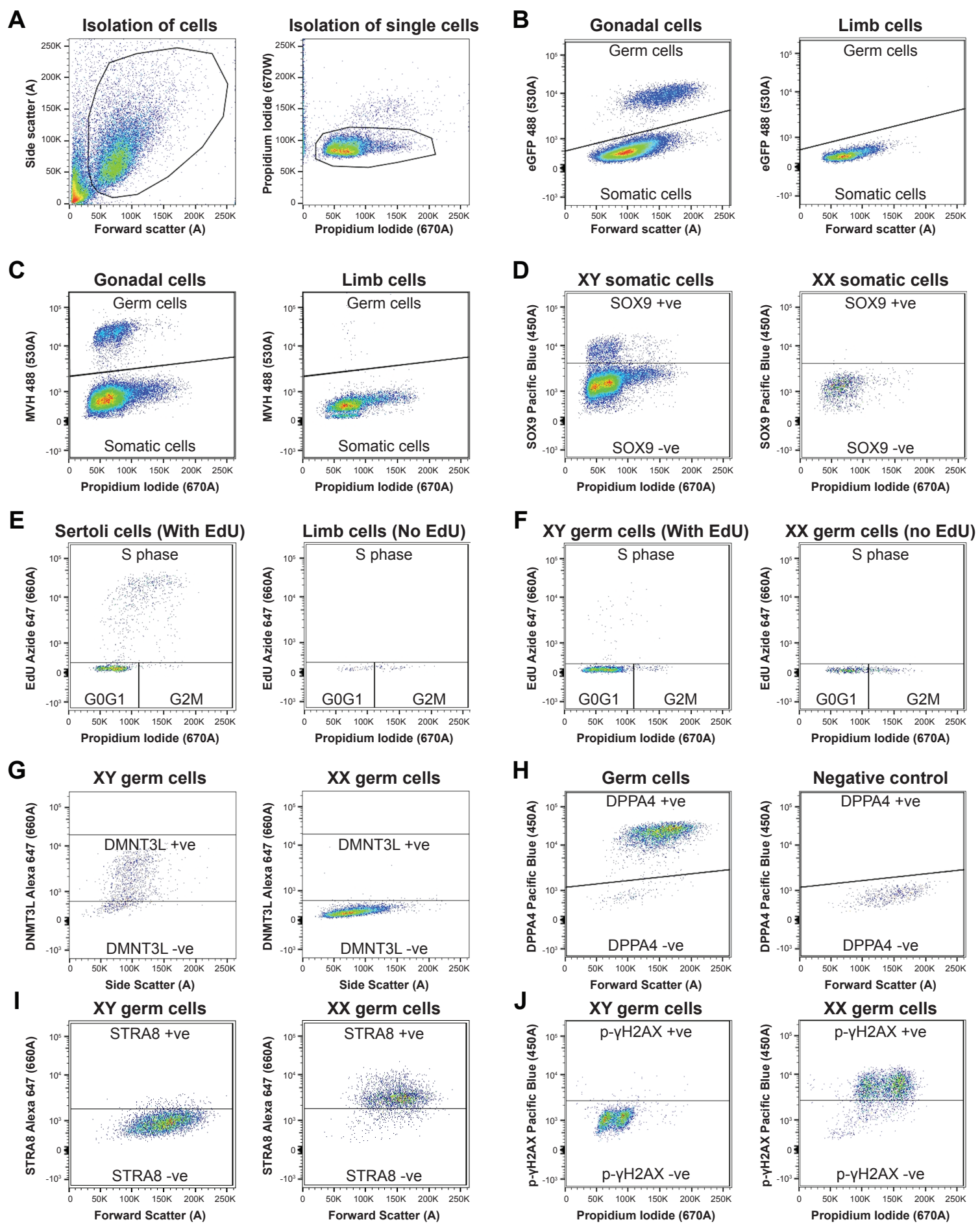

Supplement: Supplementary file 10 — Additional file 10: Figure S8. Representative plots depicting gating for antibodies used in flow cytometric analysis. A = Area, W = Width. A Gates used to separate cells from debris (left) and to isolate single cells based on propidium iodide staining (right). B Germ and somatic cells populations were identified by detecting Oct4eGFP transgene (left). E12.5 mouse limb or mesonephros cells were used as a negative control (right). C Germ and somatic cells populations were identified by detecting MVH staining (left). E12.5 mouse limb or mesonephros were used as a negative control (right). D Sertoli cells were identified based on SOX9 staining (left). XX somatic cells were used as a negative control (right). E,F Incorporation of EdU was used to identify proliferating Sertoli cells (E) or germ cells (F), with PI incorporation used to determine individual cell DNA content (left). E12.5 limb or mesonephros (E) or E12.5 XX germ cells not exposed to EdU (F) were used as a negative control for EdU (right). G DNMT3L+ germ cells identified with DNMT3L staining (left). XX germ cells were used as a negative control (right). H DPPA4+ germ cells were identified with DPPA4 staining (left). Cells not stained for DPPA4 were used as a negative control (right). I,J E12.5 + 72h XY DMSO germ cells were used as a negative control for STRA8 (I) or p-γH2AX (J) staining (left). E12.5 + 72h XX DMSO germ cells were used as a positive control (right). [file 12915_2023_1777_MOESM10_ESM.pdf]
