# Supplementary material for: FGF-independent MEK1/2 signalling in the developing foetal testis is essential for male germline differentiation in mice
Source: BMC Biol. 2023 Dec 5;21:281. doi: 10.1186/s12915-023-01777-x (PMC10696798; doi:10.1186/s12915-023-01777-x)
Supplement: Supplementary file 2 — Additional file 2: Figure S2. FGF and MEK1/2 inhibition from E12.5 does not cause sex reversal of the gonads. Analysis of XY E12.5 or XX E12.5 gonad-mesonephros tissue cultured with DMSO or 500 nM of FGFRi or MEKi for 72 h. A Immunofluorescent images demonstrating AMH and SOX9 staining. Top panel: DAPI (blue), AMH (green), SMA (red), SOX9 (cyan). Middle panel: AMH (grey). Bottom panel: SOX9 (grey). B SOX9 staining intensity in Sertoli cells determined by flow cytometry. C Immunofluorescent images demonstrating FOXL2 staining. Top panel: DAPI (blue), MVH (green), NR2F2 (red), FOXL2 (cyan). Bottom panel: FOXL2 (grey). D RNA sequencing results in isolated gonadal somatic cells following 72 h culture of key female and male gonadal somatic cell markers including Foxl2, Rspo1, Bmp2, Wnt4, Fst and Cyp26b1. Data shows the fold-change between XX E12.5 DMSO v XY E12.5 DMSO, XY E12.5 FGFRi v XY E12.5 DMSO and XY E12.5 MEKi v XY E12.5 DMSO. Plus (+) or minus (–) symbol indicates increased or decreased expression, respectively. Asterisks indicates statistical significance based on FDR<0.05 and FC >1.5. Scale bar represents 100 μm. Replicates: A,C n = 3-4, Bi n = 8-11, Bii n = 3-16, D n = 4 Statistics: Bi Welch ANOVA with Dunnett’s T3 multiple comparisons, Bii Ordinary one-way ANOVA with Tukey’s multiple comparison. In B; Intensity is relative to DMSO control sample set at 1.0. Error bars: Mean ± SEM. Significance between controls and treatment: *P<0.05, ***P<0.001. [file 12915_2023_1777_MOESM2_ESM.pdf]

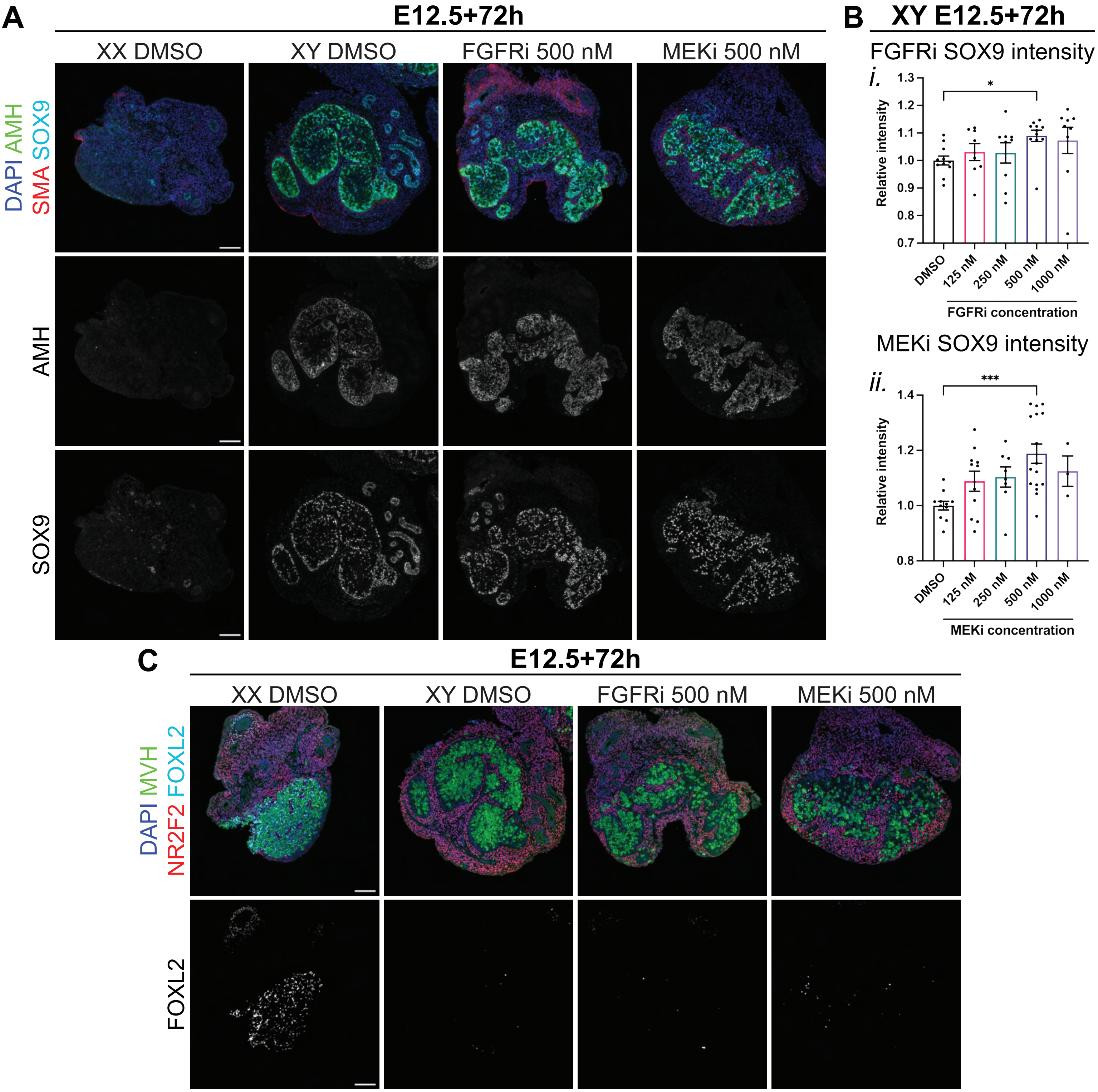

D **Isolated gonadal somatic cell RNAseq: E12.5+72h**

Genes	DMSO F v DMSO M Fold change	FGFRi M v DMSO M Fold change	MEKi M v DMSO M Fold change
Foxl2	+12.490*	-1.155	+1.497
Rspo1	+5.687*	+1.611	+1.548
Bmp2	+2.415*	-1.006	+1.316
Wnt4	+1.425	-1.864	-1.173
Fst	+8.738*	+1.158	+1.559
Cyp26b1	-9.808*	-1.197	-2.796*

*Indicates statistical significance based on FDR<0.05 and Fold Change >1.5
